# Dietary Inflammation, Gut Mycobiota, and Microbial Cross-Kingdom Associations with Cardiovascular Health in Older Adults

**DOI:** 10.3390/metabo16080578

**Published:** 2026-08-16

**Authors:** Yuchen Fu, Yuxiao Wu, Shuyue Wang, Xiaoyang An, Yong Li, Meihong Xu

**Affiliations:** 1 Department of Nutrition and Food Hygiene, School of Public Health, Peking University, Beijing 100191, China; 2111220015@stu.pku.edu.cn (Y.F.);; 2 Beijing Key Laboratory of Toxicological Research and Risk Assessment for Food Safety, Peking University, Beijing 100191, China; 3 Community Health Service Center of Wenquan Town, Beijing 100095, China; 4 Institute of Medical Technology, Peking University Health Science Center, Beijing 100191, China

**Keywords:** dietary inflammatory index, Life’s Essential 8, cardiovascular health, gut mycobiome, bacterial–fungal co-occurrence, cross-kingdom associations, zero-inflated negative binomial regression, older adults

## Abstract

**Background/Objectives:** Dietary inflammation may influence cardiometabolic health, yet the gut mycobiome and bacterial–fungal interactions remain unclear. Building upon our earlier findings and data from the TALENTs trial (Targeting Aging and Longevity with Exogenous Nucleotides) baseline data, we explored gut fungal profiles and bacterial–fungal co-occurrence patterns in relation to the Dietary Inflammatory Index (DII) and Life’s Essential 8 (LE8) in older adults. **Methods:** We enrolled 301 community residents aged 60–70 years, with 285 providing qualified fungal internal transcribed spacer (ITS) sequencing data. DII scores were derived from 3-day dietary records to reflect dietary inflammatory risk, and LE8 (integrating health behaviors including physical activity and metabolic health factors including BMI, blood lipids, blood pressure, and blood glucose) was used to assess cardiovascular health; LE8_non-diet was applied as a sensitivity measure. Fungal diversity, genus-level taxa, ecological guilds, and bacterial–fungal associations were analyzed using diversity indices, ZINB/Hurdle models, bootstrap, E-values, DIABLO analysis, and network construction. **Results:** DII showed an inverse correlation with LE8_non-diet (r = −0.130, *p* = 0.024). Fungal alpha and beta diversity did not differ significantly across DII-defined groups. Conversely, better cardiovascular status was linked to higher fungal richness, with significantly elevated Chao1 and ACE indices in the high-CVH group (both *p* < 0.05). Additionally, integrated ZINB, Hurdle, and stability analyses jointly pinpointed four candidate fungal genera that correlated with both DII and LE8. Both ZINB and DIABLO analyses consistently indicated that antagonistic interactions dominated gut bacterial–fungal associations (89.9% vs. 63.8% of negative associations, respectively), with DIABLO further revealing synchronized community-level co-variation between the two kingdoms (r = 0.325, *p* < 0.01). FUNGuild prediction further revealed saprotrophic guilds enriched in the high-CVH group and host-associated guilds in the low-CVH group, with similar patterns across DII-defined groups. **Conclusions:** This cross-sectional study reveals gut mycobiome profiles and potential bacterial–fungal co-occurrence patterns among older adults stratified by dietary inflammatory potential and cardiovascular health status, generating testable hypotheses for subsequent nutritional and metabolic research integrating lifestyle behaviors and functional health outcomes.

## 1. Introduction

Population aging has become a major public health challenge worldwide. Aging is accompanied by progressive physiological decline and is closely linked to an increased risk of cardiovascular disease (CVD), which remains one of the leading causes of morbidity and mortality among older adults [1]. Chronic low-grade inflammation critically links aging to cardiovascular dysfunction via endothelial injury, metabolic dysregulation, and atherosclerosis [2,3,4], with these disturbances reflected in circulating and tissue metabolites that serve as functional readouts of host–environment interactions. Therefore, identifying modifiable lifestyle factors related to inflammation and cardiovascular health is particularly important for prevention-oriented health management in older populations.

Diet is one of the important modifiable determinants of systemic inflammation. The Dietary Inflammatory Index (DII) was developed to quantify the overall inflammatory potential of an individual’s diet based on the intake of multiple dietary components and their literature-derived inflammatory effect scores [5]. A higher DII score indicates a more pro-inflammatory dietary pattern. Previous studies have linked higher DII to adverse cardiometabolic profiles, including hypertension, obesity, diabetes, CVD, and mortality [6]. However, most studies have focused on specific disease outcomes or individual cardiometabolic indicators, and evidence linking dietary inflammatory potential to comprehensive cardiovascular health status in older adults remains limited.

The American Heart Association’s Life’s Essential 8 (LE8) framework updates the assessment of cardiovascular health (CVH) by integrating four health behaviors (diet, physical activity, nicotine exposure, sleep health) and four health factors (body mass index, blood lipids, blood glucose, blood pressure) into a multidimensional cardiovascular score [7]. Importantly, its explicit inclusion of physical activity directly anchors LE8 to physical performance—a key healthy aging domain modifiable by nutritional and lifestyle strategies. Higher LE8 scores indicate better cardiovascular health and have been associated with lower risks of CVD events and adverse outcomes [8,9]. Because DII reflects the inflammatory potential of diet, whereas LE8 represents a multidimensional cardiometabolic health profile, examining their association may help clarify whether inflammation-oriented dietary assessment provides additional insight into cardiovascular health among older adults. Nevertheless, because LE8 itself includes a dietary component, this association should be interpreted cautiously. Analyses excluding the dietary component of LE8 are needed to assess whether any observed association extended beyond construct overlap with dietary assessment metrics.

The gut microbiome is an important interface linking diet, host metabolism, immune regulation, and cardiovascular health [10,11]. Most previous studies on the diet–microbiome–cardiovascular axis have focused primarily on bacterial communities. Gut bacterial dysbiosis may contribute to cardiovascular risk through multiple pathways, including altered bile acid metabolism [12], reduced short-chain fatty acid production [13], insulin resistance [14], lipid metabolism, and trimethylamine N-oxide-related mechanisms [15]. However, the gut ecosystem is not composed of bacteria alone. Fungi, although representing a relatively low-abundance component of the intestinal microbiome, may also participate in immune regulation, metabolic interactions, and host–microbe communication [16,17]. Previous studies have shown that the gut microbiota modulates energy metabolism, systemic inflammation, and muscle–gut signaling, thereby influencing physical performance and exercise adaptation [18,19], yet their role in the relationship between dietary inflammatory potential and cardiovascular health remains insufficiently characterized.

Diet may influence gut bacterial community composition and ecological functions by altering available substrates, gut environmental conditions, and bacterial–fungal interactions [20]. Cross-kingdom bacterial–fungal associations may shape gut ecosystem stability through nutrient competition, metabolic cooperation, niche occupation, and immune-related interactions. Such co-occurrence patterns may be particularly relevant under inflammatory or metabolic disturbances [21,22]. In addition, fungal abundance data are often sparse and zero-inflated, which may limit the sensitivity of conventional association models and increase the uncertainty of genus-level findings. Zero-inflation-aware models and sensitivity analyses may therefore provide exploratory evidence for identifying candidate fungal signals.

Using baseline data from the TALENTs trial (Targeting Aging and Longevity with Exogenous Nucleotides), we aimed to assess DII associations with total LE8 and diet-excluded LE8, and to describe gut mycobiome and bacterial–fungal co-occurrence patterns among older adults stratified by DII/LE8. Our findings provide exploratory evidence to inform future research on potential links between dietary inflammatory potential, gut mycobiome, cardiometabolic health, and physical function in older adults.

## 2. Materials and Methods

### 2.1. Study Design and Participants

This cross-sectional study was based on baseline survey data from the TALENTs trial, a human clinical study designed to investigate the anti-aging effects of exogenous nucleotides in older adults [23]. The present analysis extends our previously published bacterial microbiome study by incorporating gut mycobiome profiling and bacterial–fungal cross-kingdom co-occurrence analysis [24].

A total of 301 community-dwelling older adults aged 60–70 years were included. Baseline data included demographic characteristics, lifestyle factors, chronic disease history, dietary intake, physical examination measurements, biochemical indicators, and fecal samples. Detailed information regarding the TALENTs trial design and baseline assessment procedures has been described previously [23].

Fresh fecal samples were collected at baseline, transported under cold-chain conditions within 24 h and stored at −80 °C until analysis. After quality control, 285 samples were available for fungal internal transcribed spacer (ITS) sequencing analysis. Bacterial data used for cross-kingdom analysis were obtained from the previously published 16S rRNA sequencing dataset from the same baseline population [24]. The original TALENTs trial was conducted in strict accordance with the Declaration of Helsinki, approved by the Biomedical Ethics Committee of Peking University (approval No. IRB00001052-21114), and registered at ClinicalTrials.gov (registration No. NCT05243108). All participants provided written informed consent. The present study is a secondary analysis of de-identified baseline survey data and did not involve additional interventions.

### 2.2. DII and LE8 Assessment

Dietary intake was assessed using a 3-day photo-assisted dietary assessment method, as described in the TALENTs study protocol [23]. Briefly, participants received standardized instructions prior to data collection and were asked to maintain their usual eating habits throughout the recording period. They photographed all foods and beverages before and after consumption on three consecutive days. Trained researchers then estimated portion sizes and nutrient intakes from these images, and average daily dietary intake was calculated for subsequent analyses. Given that the assessment was based on only three consecutive days, the resulting dietary variables were interpreted as reflecting short-term intake during the baseline period rather than long-term habitual dietary patterns.

The Dietary Inflammatory Index (DII) is an objective measure, and the calculation of DII follows the method proposed by Shivappa et al. [25]. Specifically, for the 22 dietary components included in the calculation (energy, protein, carbohydrates, total fat, cholesterol, saturated fatty acids, monounsaturated fatty acids, polyunsaturated fatty acids, dietary fiber, folic acid, f vitamins A, B1, B2, B3, B6, B12, Cand E, as well as zinc, magnesium, iron, and selenium), the individual’s daily intake is first subtracted from the overall mean and divided by the overall standard deviation to obtain a Z-value. This Z-value is then converted into a cumulative proportion, multiplied by 2, and subtracted by 1. Finally, this value is multiplied by the corresponding component-specific inflammation effect scores. The comprehensive DII score is ultimately derived by summing all component-specific scores. A higher DII score indicates a more pro-inflammatory dietary pattern. The number of DII components was constrained by the dietary variables available in the original TALENTs baseline assessment. Several components that are generally consumed at low levels in the general population, such as eugenol, isoflavones, and capsaicin, were not included in the index calculation. Previous studies [5,24] have demonstrated that the DII score calculated based on a reduced set of components remains valid. Participants were categorized into tertiles according to their DII scores, namely the low-DII (LDII), middle-DII (MDII), and high-DII (HDII) groups, following the analytical strategy commonly adopted in previous DII-based studies.

Cardiovascular health was assessed via the LE8 score developed by the American Heart Association [26]. The LE8 metric comprises four health behaviors—the dietary component, physical activity (an important behavioral factor for maintaining and improving physical function [27]), tobacco exposure, and sleep health—and four health factors—namely body mass index (BMI), blood lipids (quantified as Non-HDL cholesterol), blood pressure (evaluated via systolic and diastolic BPs), and blood glucose (measured as FPG or HbA1c)—which together form a multidimensional cardiovascular score (Table S1). Each component was scored from 0 to 100, and the total LE8 score was calculated as the arithmetic mean of the eight components. A higher LE8 score indicates better cardiovascular health. Participants were further categorized into low (0–49), moderate (50–79), and high (80–100) cardiovascular health (CVH) groups according to the LE8 scoring criteria. Because the LE8 score includes a dietary component and therefore partially overlaps with DII, an additional LE8 score excluding the dietary component, referred to as LE8_non-diet, was calculated and used as a sensitivity outcome.

### 2.3. ITS Sequencing and Taxonomic Annotation

Fecal fungal communities were characterized by high-throughput sequencing of the internal transcribed spacer (ITS) region. Genomic DNA was extracted from fecal samples, and the ITS1-5.8S-ITS2 region was amplified using primers ITS1F and ITS2R. PCR products were examined by 2% agarose gel electrophoresis, purified, quantified, and sequenced on the Illumina MiSeq platform (Illumina, San Diego, CA, USA) using the PE300 strategy. Raw reads were quality-filtered using fastp (v0.20.0) and subsequently merged using FLASH (v1.2.11) based on overlapping paired-end sequences; valid sequences were assigned to samples based on sample-specific barcodes and primers.

Subsequently, valid sequences were processed using QIIME 2 (v2024.5). Denoising was performed with DADA2 to generate amplicon sequence variants (ASVs) and an abundance table. Taxonomic classification was then assigned using the sklearn classifier against the UNITE database (v10, 4 April 2024). The final outputs included representative sequences, a feature table, and taxonomy assignments for downstream diversity analyses.

A total of 20,869,395 valid sequences were retained from 285 samples (per-sample range: 40,480–143,658; median: 72,162). The Q30 quality score ranged from 91.95% to 99.37% across samples (median: 98.39%), indicating high sequencing quality. A total of 7871 ASVs were obtained after quality filtering and chimera removal.

### 2.4. Statistical Analysis

Continuous variables were described as mean ± standard deviation or median with interquartile range, and categorical variables were described as frequencies and percentages. Spearman correlation analysis was used to evaluate the crude associations of DII with total LE8 score and LE8_non-diet score, followed by multivariable linear regression. The adjusted model included covariates of demographics, socioeconomic status, lifestyle factors, digestive system diseases, and constipation status.

Alpha diversity (Chao1, ACE, Shannon) was calculated at the ASV level and compared across groups using the Kruskal–Wallis test. Beta diversity was assessed via unweighted and weighted UniFrac distances, visualized by principal coordinates analysis (PCoA), and tested by permutational multivariate analysis of variance (PERMANOVA) (R package vegan v2.7.5).

For genus-level DII/LE8 associations, zero-inflated negative binomial (ZINB) regression was performed using the pscl package (v1.5.9; zeroinfl function). False discovery rate (FDR) correction (Benjamini–Hochberg, q < 0.05) was applied independently to DII and LE8 models; genera significant in both were considered candidates. Robustness was evaluated using hurdle models (pscl) and bootstrap resampling (1000 iterations; boot package v1.3.32); genera with significant and directionally consistent associations in >50% of resamples were deemed stable. E-values were calculated using a custom function based on VanderWeele & Ding [28] to quantify sensitivity to unmeasured confounding; larger E-values indicate greater robustness against potential unmeasured confounders. These analyses do not imply causal inference.

For cross-kingdom co-occurrence, bacterial genera from prior 16S data [24] were integrated with fungal genera (detection > 5%). Pairwise associations were tested using ZINB regression via the pscl package (v1.5.9) with FDR correction. Additionally, following the Data Integration Analysis for Biomarker discovery using Latent cOmponents (DIABLO) methodology [29], multi-omics integrative analysis was performed using the R package mixOmics (v6.10.9). Genus-level relative abundance data for bacteria and fungi underwent 5% prevalence filtering and centered log-ratio transformation; the design matrix (with candidate values ranging from 0 to 1) was incorporated as a core parameter into a grid search, with the number of latent components fixed at 2 (number of groups minus 1, following the methodological recommendation [29]) and the number of features to retain per component per omics searched from 5 to 30 (step = 5); optimal parameters were determined by 5-fold cross-validation repeated 5 times minimizing the average balanced error rate. The final model automatically selected features via an L1 sparsity penalty. After model fitting, Pearson correlation was performed on the first-component scores of both omics blocks to assess global co-variation between bacterial and fungal communities, and subsequent pairwise Pearson correlation analyses were performed based on the selected features.

Fungal ecological functions were predicted by the FUNGuild database implemented in the R package FUNGuildR (v0.3.0) and compared across DII/CVH groups, interpreted as exploratory clues. All analyses used R 4.5.2; two-sided *p* < 0.05 was considered significant unless otherwise specified.

## 3. Results

### 3.1. Demographic Characteristics and DII–LE8 Association

**Figure 1:**
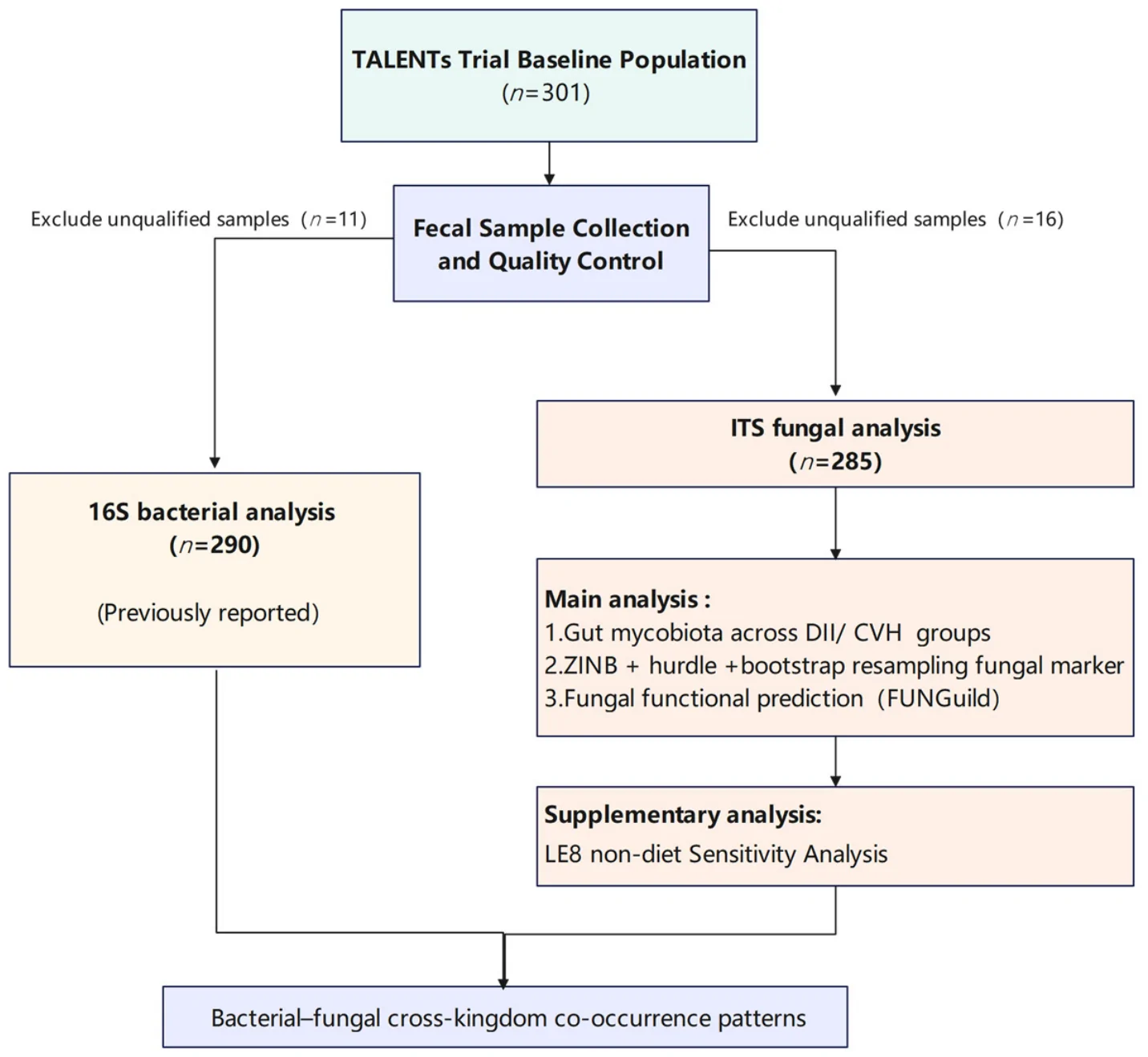
Study flowchart.

The participant flow and analytical framework of the present extension analysis are shown in Figure 1.

The primary association between DII and total LE8 score in the full TALENTs baseline sample has been reported previously [24], and the baseline characteristics of the study population are summarized in Table 1. Briefly, higher DII was associated with lower total LE8 score. The present analysis focused on participants with qualified ITS sequencing data and examined gut mycobiome features and bacterial–fungal cross-kingdom co-occurrence patterns in relation to dietary inflammatory potential and cardiovascular health.

Because the LE8 score includes a dietary component and may partially overlap with DII, LE8_non-diet was additionally examined as a sensitivity outcome. Normality was assessed using the Shapiro–Wilk test: LE8 (*p* = 0.118) and LE8_non-diet (*p* = 0.220) were normally distributed, while total DII significantly deviated from normality (*p* < 0.001). Accordingly, Spearman’s rank correlation was used for all correlation analyses. DII was inversely correlated with LE8_non-diet in the crude correlation analysis (*ρ* = −0.130, *p* = 0.024). In multivariable linear regression, the association between DII and LE8_non-diet was attenuated after adjustment for demographic, socioeconomic, lifestyle, digestive system disease, and constipation-related covariates (β = −1.018, *p* = 0.0763) (Table S2), indicating that the association between DII and total LE8 may be partially driven by the dietary component of LE8.

### 3.2. Gut Mycobiota Across DII Groups

**Figure 2:**
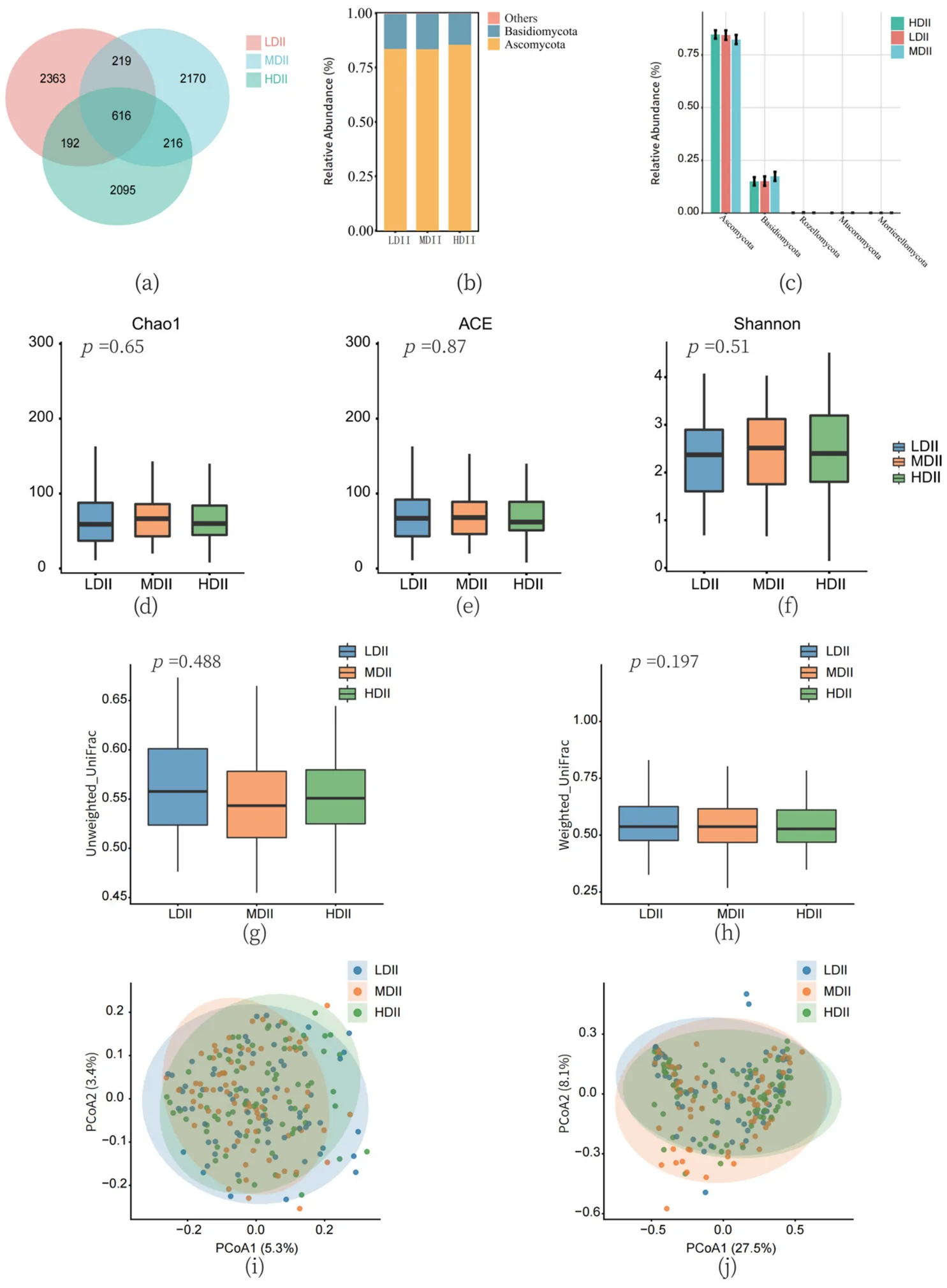
Gut mycobiome composition and diversity across DII groups (*n* = 285). (**a**) Venn diagram illustrating the distribution of ASVs across three DII groups; (**b**) Community composition of intestinal fungi at phylum level among the three DII groups; (**c**) Comparison of the top five fungal phyla by relative abundance at phylum level across the three DII groups; (**d**) Comparison of Chao1 index; (**e**) Comparison of ACE index; (**f**) Comparison of Shannon index; (**g**) Unweighted UniFrac distance comparison; (**h**) Weighted UniFrac distance comparison; (**i**) PCoA based on unweighted UniFrac distance; (**j**) PCoA based on weighted UniFrac distance.

Fungal sequencing data from 285 participants were used to compare gut mycobiome composition across DII groups. At the ASV level, the low-DII, middle-DII, and high-DII groups contained 3390, 3221, and 3119 ASVs, respectively, with 616 ASVs shared across all three groups (Figure 2a).

At the phylum level, 13 fungal phyla were detected. The fungal community was dominated by Ascomycota and Basidiomycota, with mean relative abundances of 83.79% and 15.88%, respectively; together, these two phyla accounted for 99.67% of total fungal abundance (Figure 2b,c). The remaining fungal phyla were rare and collectively accounted for less than 1% of the total community. No fungal phylum showed a statistically significant difference in relative abundance across DII groups (all *p* > 0.05).

Alpha diversity analysis showed no significant differences in Chao1, ACE, or Shannon indices among the three DII groups (all *p* > 0.05; Figure 2d–f). Beta diversity analysis based on unweighted UniFrac distance showed no significant separation of fungal community structure by DII group (PERMANOVA R^2^ = 0.0088, *p* = 0.488; Figure 2g,i). Similar results were observed using weighted UniFrac distance (PERMANOVA R^2^ = 0.0107, *p* = 0.197; Figure 2h,j). Detailed results are provided in Table S3.

### 3.3. Gut Mycobiota Across CVH Groups

**Figure 3:**
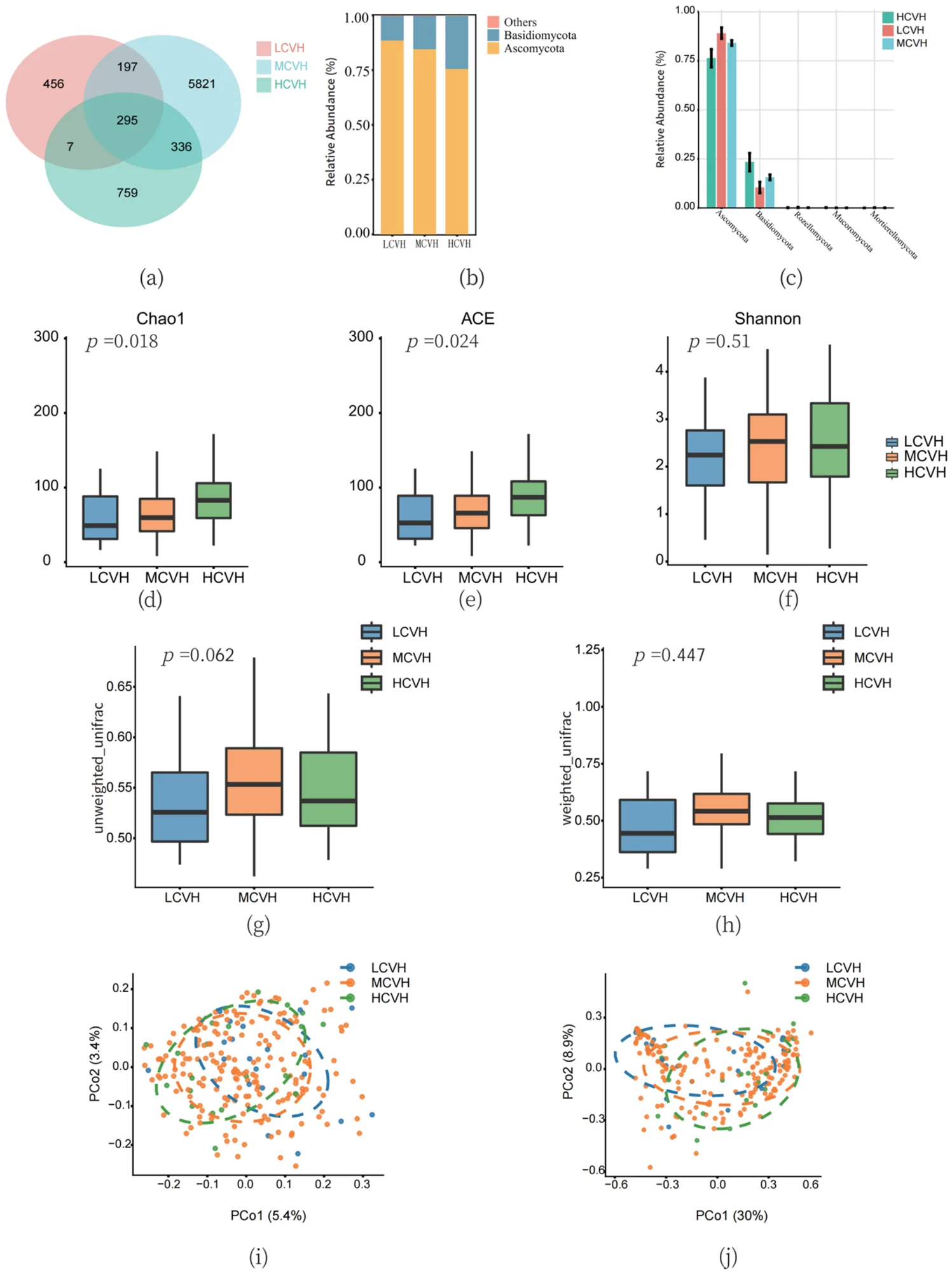
Gut mycobiome composition and diversity across CVH groups *(n* = 285). (**a**) Venn diagram illustrating the distribution of ASVs across three CVH groups; (**b**) Community composition of intestinal fungi at phylum level among the three groups; (**c**) Comparison of the top five fungal phyla by relative abundance at the phylum level among the three groups; (**d**) Comparison of Chao1 index; (**e**) Comparison of ACE index; (**f**) Comparison of Shannon index; (**g**) Unweighted UniFrac distance comparison; (**h**) Weighted UniFrac distance comparison; (**i**) PCoA based on unweighted UniFrac distance; (**j**) PCoA based on weighted UniFrac distance.

Participants were further categorized into low, moderate, and high cardiovascular health groups according to LE8 score. At the phylum level, Ascomycota and Basidiomycota remained the dominant fungal phyla across all cardiovascular health groups, together accounting for more than 99% of total fungal abundance (Figure 3b,c). Four fungal phyla, including Ascomycota, Basidiomycota, Entorrhizomycota, and Chytridiomycota, showed nominally significant differences in relative abundance across cardiovascular health groups (*p* < 0.05).

Alpha diversity analysis showed significant differences in fungal richness across CVH groups. The Chao1 and ACE indices were higher in the high-CVH group than in the moderate- and low-CVH groups (*p* < 0.05; Figure 3d,e). In contrast, the Shannon index did not differ significantly among groups (Figure 3f).

Beta diversity analysis showed no significant separation of fungal community structure across CVH groups. The unweighted UniFrac-based PERMANOVA result was not statistically significant (R^2^ = 0.0102, *p* = 0.062; Figure 3g,i), and weighted UniFrac analysis also showed no significant difference (R^2^ = 0.0087, *p* = 0.447; Figure 3h,j). Detailed results are summarized in Table S3.

### 3.4. Genus-Level Fungal Associations Identified by ZINB Models

**Figure 4:**
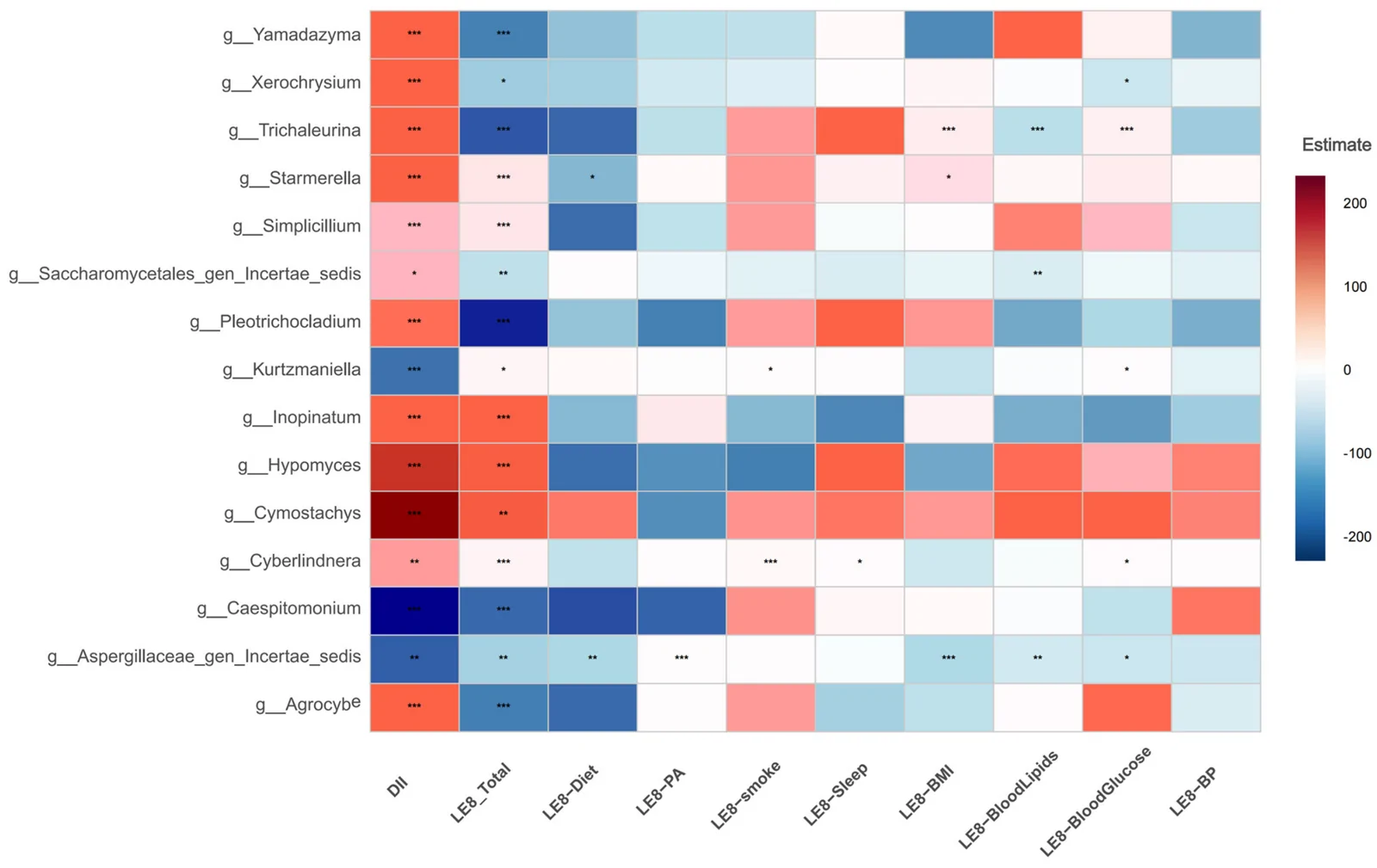
Heatmap of fungal genus-level associations with DII and LE8 scores. Color gradient reflects ZINB regression coefficients (blue = negative, red = positive). Significance markers (* FDR < 0.05, ** FDR < 0.01, *** FDR < 0.001) are based on FDR correction. Rows: clustered fungal genera; columns: DII, LE8 total score, and the eight LE8 components. White indicates missing values from non-convergent models.

Given the high sparsity and zero inflation of fungal abundance data, ZINB regression was applied as a zero-inflation-aware analysis. The full ZINB results are listed in Table S4. Among 620 fungal genera, 39 were significantly associated with DII and 78 were significantly associated with total LE8 score after FDR correction. The intersection of these results identified 15 candidate fungal genera associated with both DII and LE8 (Figure 4).

Sensitivity analysis using the Hurdle model identified a total of 27 genera that were significantly associated with both DII and LE8. Among these, 6 genera overlapped with those identified by the ZINB model and showed regression coefficient directions consistent with the ZINB estimates (Table S4D). In the bootstrap analysis with 1000 iterations, four genera-Kurtzmaniella, Aspergillaceae_gen_Incertae_sedis, Cyberlindnera, and Saccharomycetales_gen_Incertae_sedis—exhibited stability proportions > 0.5 for both DII and LE8 predictors (Table S4E).

For these four genera, we calculated the E-values for the point estimates and the lower bounds of their 95% confidence intervals (Table S4F). All DII-associated point estimates had E-values greater than 2 (range: 2.49–3.02), with the lower confidence interval (LCI) E-values ranging from 1.55 to 4.43. For total LE8, the point estimate E-values ranged from 1.25 to 1.39, and the LCI E-values ranged from 1.19 to 1.51.

### 3.5. Bacterial–Fungal Cross-Kingdom Co-Occurrence Patterns

**Figure 5:**
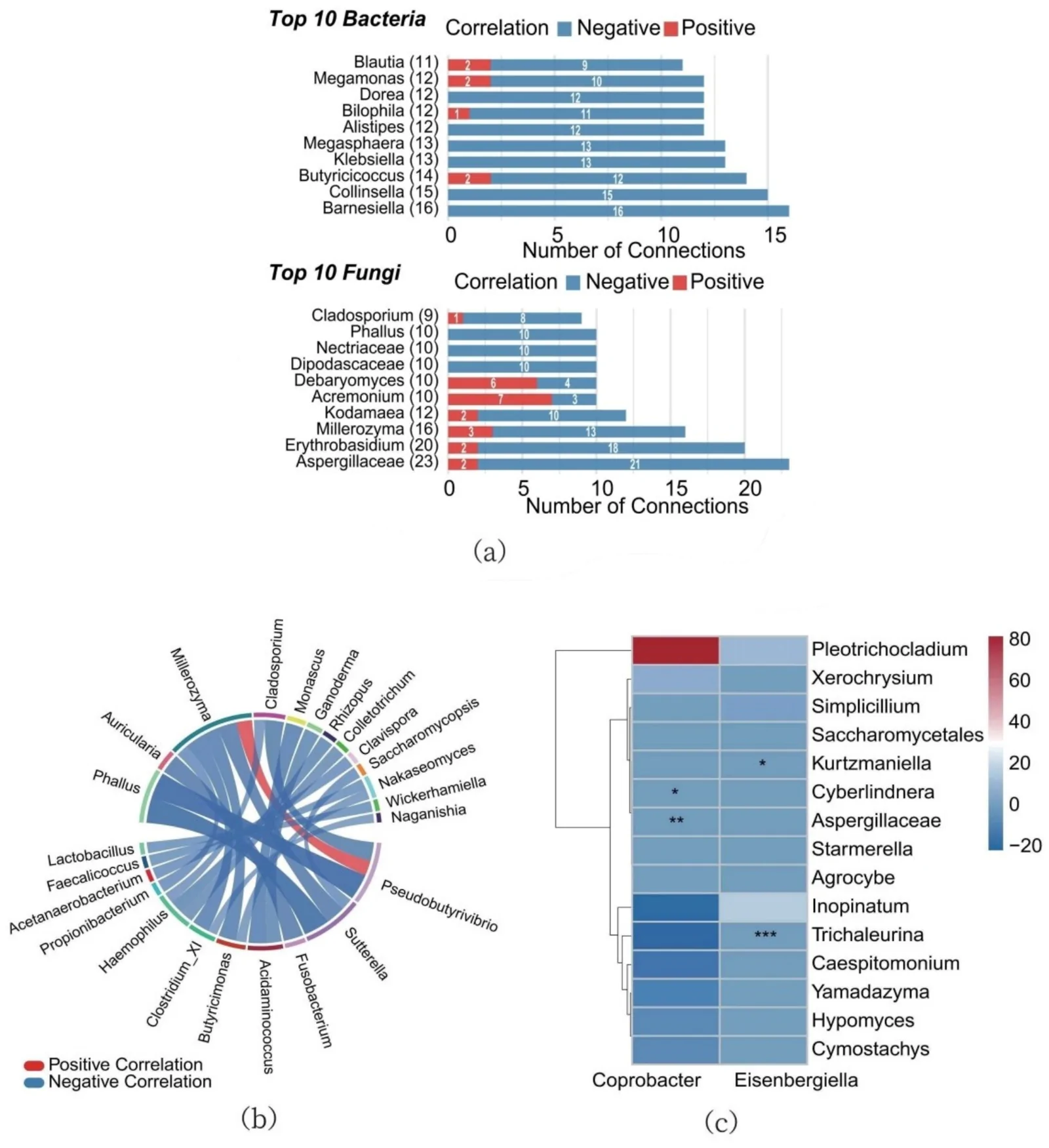
Bacterial–fungal cross-kingdom co-occurrence patterns. (**a**) Top connected bacterial and fungal nodes; (**b**) chord diagram showing strongly associated bacterial–fungal pairs; (**c**) heatmap of bacterial–fungal associations among taxa associated with both DII and LE8 (* FDR < 0.05; ** FDR < 0.01; *** FDR < 0.001).

With a 5% detection cutoff, 57 bacterial and 96 fungal genera were included for cross-kingdom co-occurrence analysis. ZINB modeling revealed 417 FDR-adjusted significant bacterial–fungal associations (FDR < 0.05; Table S5), 89.9% of which were negative. Among fungal genera, Aspergillaceae_gen_Incertae_sedis, Erythrobasidium, and Millerozyma showed relatively high node degrees. Among bacterial genera, Barnesiella, Collinsella, and Butyricicoccus showed relatively high node degrees (Figure 5a). The strongest negative associations included Pseudobutyrivibrio–Phallus, Sutterella–Phallus, and Fusobacterium–Auricularia (Figure 5b). Among taxa associated with both DII and LE8, four significant negative bacterial–fungal associations were identified (Figure 5c). Coprobacter was negatively associated with Cyberlindnera and Aspergillaceae_gen_Incertae_sedis, whereas Eisenbergiella was negatively associated with Kurtzmaniella and Trichaleurina.

**Figure 6:**
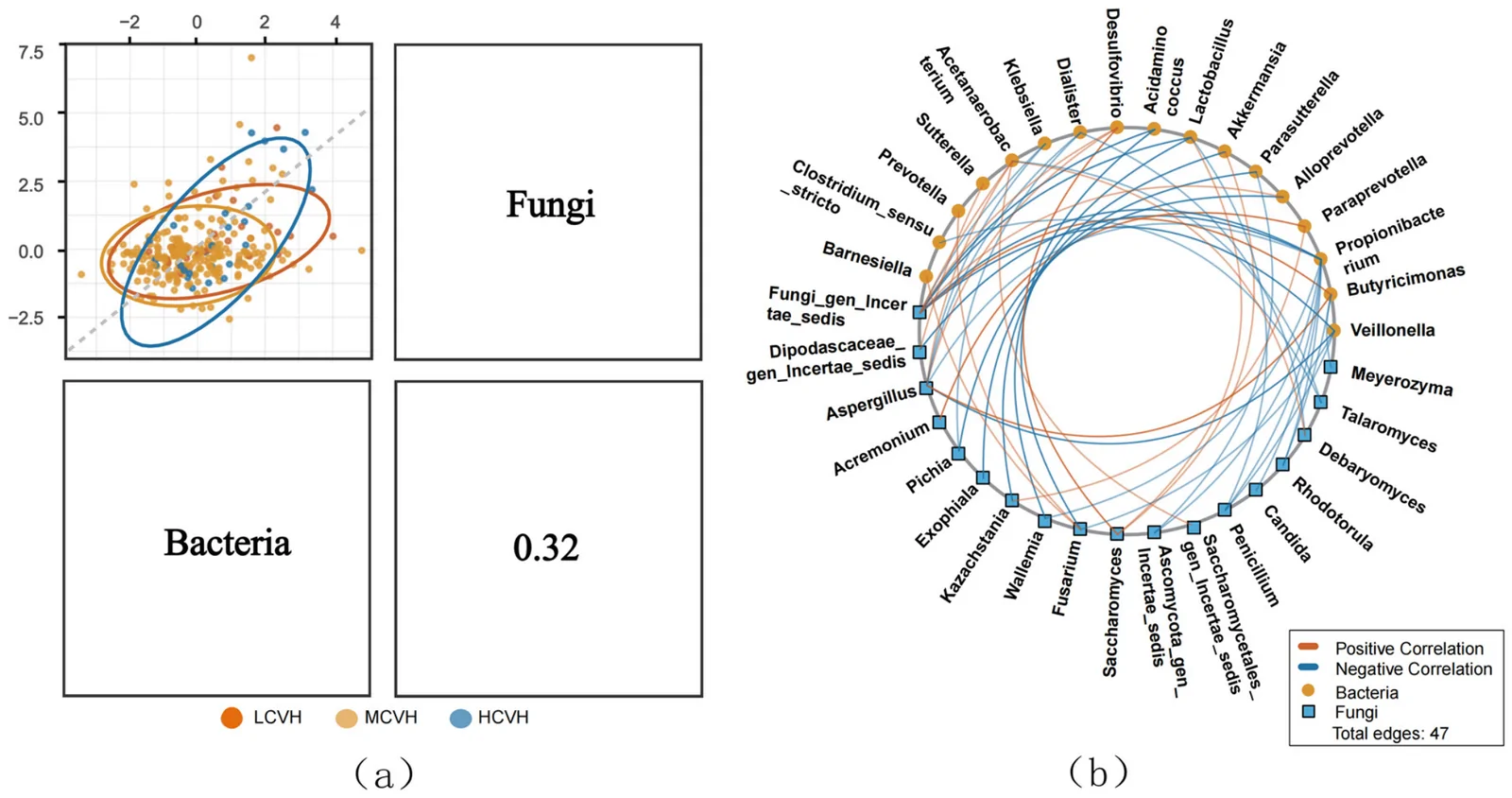
DIABLO-based integration of bacterial and fungal cross-kingdom co-variation patterns. (**a**) Global correlation between DIABLO component 1 of bacteria and fungi (red: LCVH; orange: MCVH; blue: HCVH) (r = 0.325, *p* < 0.01); (**b**) Circular plot of significant bacteria–fungi associations based on DIABLO-selected features. Red links: positive correlations; blue links: negative correlations; link thickness is proportional to the absolute correlation coefficient.

Further supervised multi-omics integration using DIABLO achieved an average balanced error rate of 0.4404 (minimum BER = 0.4298) with design = 0.7 and ncomp = 2, retaining 17 bacterial and 18 fungal features in total. Sample scores on the first component of bacteria and fungi showed a significant positive correlation (r = 0.325, *p* < 0.01), indicating global co-variation between the two kingdoms (Figure 6a). Based on the L1-sparsity-selected features, subsequent Pearson correlation analysis identified 47 significant associations (*p* < 0.05; Figure 6b, Table S5B), of which 63.8% were negative.

### 3.6. Fungal Functional Prediction

**Figure 7:**
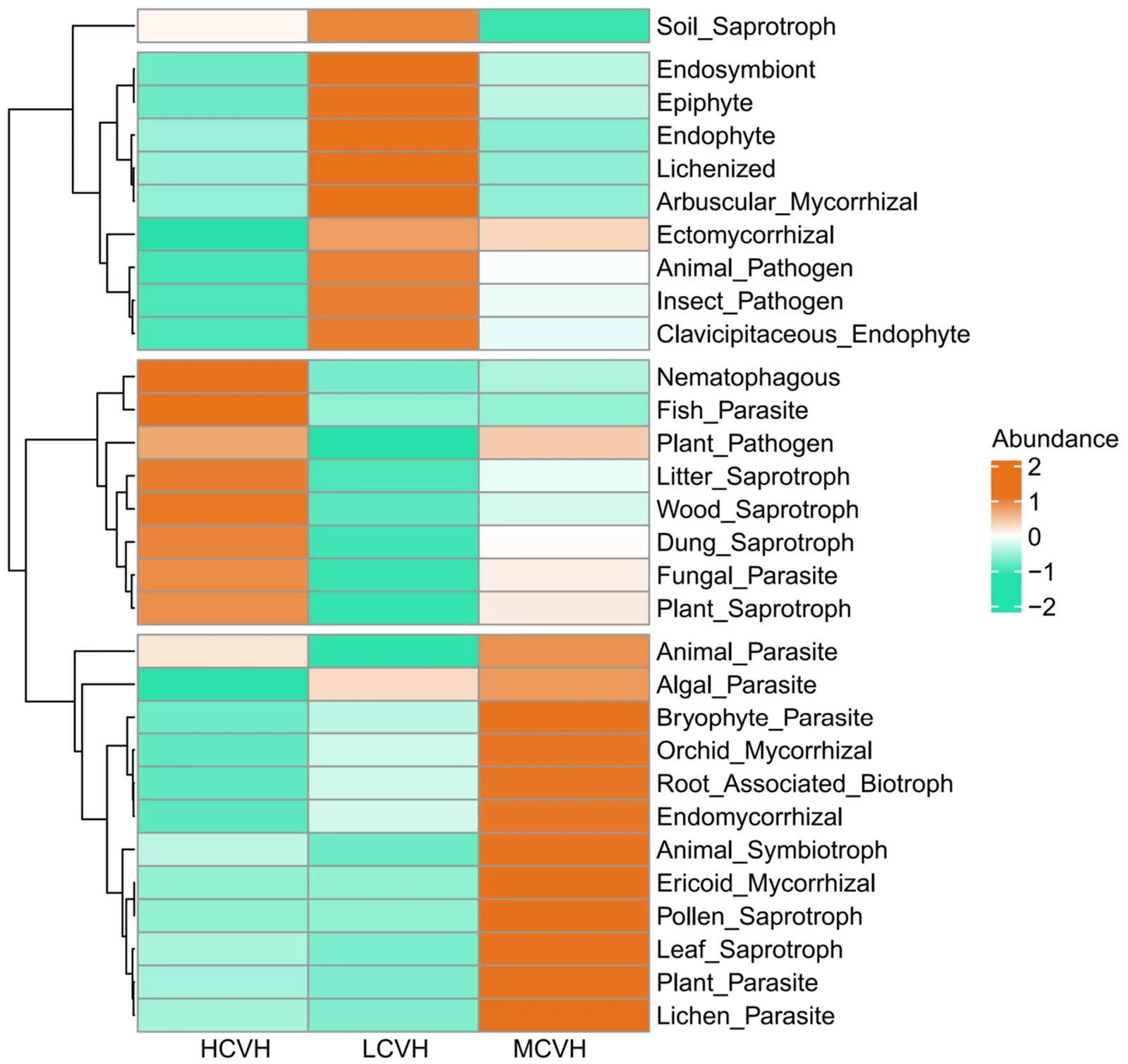
FUNGuild-based prediction of fungal ecological functions across cardiovascular health groups. The heatmap shows Z-score-standardized abundances (orange: high; cyan: low), with hierarchical clustering applied using Euclidean distance and complete linkage.

Fungal ecological functions were predicted using FUNGuild based on taxonomic annotation. Comparisons across CVH groups showed differences in the relative abundance of several predicted ecological guilds (Figure 7 and Table S6). The high-CVH group showed higher relative abundance of several saprotrophic guilds, including Litter_Saprotroph, Wood_Saprotroph, and Dung_Saprotroph. Lower relative abundance was observed for several host-associated or symbiotrophic guilds, including Endosymbiont, Epiphyte, Lichenized, and Animal_Symbiotroph. Differences in predicted ecological guild profiles were also observed across DII-defined groups (Table S6).

## 4. Discussion

In this extension analysis of the TALENTs baseline data, we characterized gut mycobiome features and bacterial–fungal cross-kingdom co-occurrence patterns related to dietary inflammatory potential and multidimensional cardiovascular health in older adults. Building on that work, the present study focused on newly generated gut mycobiome data and integrated them with the previously published bacterial dataset [24] to explore fungal features and bacterial–fungal ecological patterns.

The sensitivity analysis showed that the inverse association between DII and LE8_non-diet was attenuated after multivariable adjustment, suggesting that the DII–total LE8 association partly reflects the dietary component of LE8. Thus, DII and LE8 should not be treated as fully independent constructs. We explicitly acknowledge that our findings reflect shared or intertwined exposures rather than causal pathways. DII should be viewed as a complementary inflammation-oriented dietary indicator, whereas LE8 evaluates a broader cardiovascular health construct that integrates physical activity-related behaviors and metabolic dimensions. The relatively narrow DII range in this population may explain the modest effect size observed, as restricted exposure variability can attenuate associations. From a public health perspective, however, even modest dietary shifts may be meaningful if sustained over time and adopted by a substantial proportion of the population—particularly for older adults, for whom dietary modification is a feasible non-pharmacological strategy to support cardiometabolic health. Previous epidemiological studies have linked higher DII with hypertension and adverse cardiometabolic outcomes [6,30], and long-term evidence suggests that lower energy-adjusted DII is associated with reduced cardiovascular and all-cause mortality [31]. Recent population-wide phenome analyses have also reported associations between diet-induced inflammation and a broad range of health outcomes, particularly involving circulatory and metabolic systems [32]. These findings support inflammation-oriented dietary assessment in understanding cardiometabolic health in older adults, though its independent association with non-dietary LE8 was modest in this population. The ecological implications of the mycobiome and cross-kingdom associations require validation in cohorts with broader DII distributions.

The gut mycobiome analysis showed limited differences across DII groups. Fungal alpha diversity (Chao1, ACE, Shannon), beta diversity, and dominant phylum-level composition did not differ significantly according to DII tertiles (all *p* > 0.05). Notably, this negative finding is itself informative: it contrasts with the previously published bacterial analysis [24] in the same population, where significant differences were observed across DII groups for Chao1 (*p* = 0.0088), ACE (*p* = 0.009), and beta diversity (Unweighted and Weighted UniFrac: both *p* < 0.001), although Shannon diversity was comparable (*p =* 0.89). Rather than defining an “optimal” mycobiome composition, the present analysis describes how gut fungal diversity varies across DII-defined dietary inflammatory gradients within this population. These quantitative comparisons suggest that gut fungal communities in this population showed weaker associations with DII-defined dietary inflammatory gradients than bacterial communities. This finding is consistent with the general understanding that bacterial communities respond rapidly or at an early stage to dietary changes [33,34], whereas fungal communities may show greater inter-individual variability and lower abundance [35], making dietary exposure-related patterns harder to detect in moderate-sized population studies. The human gut mycobiome is usually a low-biomass component of the intestinal ecosystem [36], and studies from the Human Microbiome Project and subsequent population-level analyses have shown that fungal taxa are often sparsely distributed and highly variable across individuals [37]. These features may explain why DII-based dietary grouping did not show clear fungal community separation, and they also highlight the importance of using statistical approaches that are appropriate for sparse mycobiome data. Taken together, our results provide a reference for future studies investigating diet–mycobiome associations and underscore the need for specialized analytical strategies in mycobiome research.

In contrast, cardiovascular health groups showed more evident differences in fungal richness. Participants with higher LE8 scores had higher Chao1 and ACE indices, suggesting that better cardiovascular health may be associated with greater fungal richness. However, Shannon diversity and beta diversity analyses did not show clear separation, suggesting that the observed association was mainly related to richness rather than broad restructuring of the fungal community. This result is compatible with emerging evidence that gut mycobiome features are associated with cardiovascular health and host metabolic phenotypes. For example, a nationwide Chinese cohort study reported that higher gut fungal richness was associated with lower risk of cardiometabolic diseases, while overall community structure showed no clear separation between health groups [38], which is consistent with our observation that the LE8-related mycobiome signal was primarily driven by richness rather than broad community restructuring. LE8 integrates diet, physical activity, nicotine exposure, sleep, body weight, lipids, glucose, and blood pressure, and may therefore better reflect the overall host metabolic and inflammatory environment that shapes fungal ecological niches. These results suggest that integrated cardiovascular health status—capturing both metabolic and behavioral factors—may be linked not only to bacterial profiles but also to broader multi-kingdom microbial ecology in the context of host metabolism and physical function.

Given the high sparsity and zero-inflation of fungal abundance data, we applied ZINB regression, supplemented by Hurdle models and bootstrap resampling, to identify candidate fungal genera associated with both DII and LE8. The Hurdle model agreed with the ZINB model for 6 of 15 genera, and bootstrap stability analysis pinpointed four genera with high replication stability (>50%) for both exposures, designating them as relatively reliable candidate signals. Among these, two represent unclassified taxa (Aspergillaceae_gen_Incertae_sedis and Saccharomycetales_gen_Incertae_sedis). While related species within these higher-level taxa have been previously linked to cardiac infections and cardiomyocyte injury [39,40], these indirect findings do not directly apply to the unclassified taxa identified here. The E-value results further support cautious interpretation of the genus-level findings. For DII, E-values for the four bootstrap-supported genera were greater than 2, suggesting that the associations were robust to unmeasured confounding. However, for LE8, E-values ranged from 1.25 to 1.39, indicating that the LE8-related associations were susceptible to unmeasured confounding. This concern is particularly relevant because several factors that may influence the gut mycobiome—such as recent antifungal or antibiotic use, probiotic intake, oral health status, and short-term dietary fungal exposure—were not fully captured in the original baseline survey. Moreover, many genera identified here have been more frequently documented in environmental, food fermentation [41], edible fungi [42], or soil contexts [43] than in human intestinal ecosystems. Such studies offer useful functional clues but cannot substitute for direct human gut validation. Therefore, our genus-level findings constitute a focused set of exploratory candidates that warrant future investigation using shotgun metagenomics, metatranscriptomics, metabolomics, culture-based approaches, and longitudinal sampling to determine their relevance to human health.

The extensive bacterial–fungal cross-kingdom co-occurrence patterns identified in this study provide a systems-level perspective on gut microbial ecology beyond individual taxa. Pairwise association testing based on ZINB modeling, DIABLO global co-variation, and DIABLO L1-based feature selection addressed different scales and objectives, collectively depicting a more comprehensive landscape of cross-kingdom interactions. ZINB identified 417 associations (89.9% negative), consistent with recent metagenomic evidence linking gut bacteria and fungi through dietary nutrient competition and ecological constraints [44]. DIABLO revealed a significant positive correlation on the first component (r = 0.325), indicating synchronized community-level variation between bacterial and fungal assemblages. Association analyses performed on L1-sparsity-selected core features from DIABLO identified 47 associations (63.8% negative), suggesting that competitive interactions also prevail among core microbes most relevant to host phenotypes. The three approaches are complementary: ZINB maps broad pairwise networks, DIABLO global scores reflect community-level collective behavior, and L1-selected associations pinpoint core hubs coupled with host phenotypes [29]. Together, they elucidate a complex dynamic wherein bacteria and fungi exhibit widespread competitive exclusion while maintaining coordinated responses at higher levels. The substantial difference in the number of significant associations identified by ZINB versus DIABLO is partly attributable to their distinct scopes: ZINB tests all 57 × 96 pairwise combinations, whereas DIABLO restricts analysis to the 17 × 18 L1-selected core features. Thus, these estimates reflect different resolutions rather than being directly comparable.

The cross-kingdom analysis also identified several highly connected bacterial and fungal nodes, suggesting that some taxa may occupy central positions in the observed bacterial–fungal association network. The gut mycobiome is increasingly recognized as an integral part of the broader microbiome, interacting with bacteria during community assembly and potentially influencing immune and metabolic homeostasis [45]. Reviews and experimental work have suggested that fungi and bacteria may influence each other through nutrient exchange or competition, biofilm formation, immune modulation, and changes in mucosal ecological niches [46].

Despite these structural links, the DIABLO model achieved only moderate discriminative accuracy in distinguishing CVH groups (BER = 0.4404; minimum BER = 0.4298), which outperformed random guessing (BER = 0.5) but left substantial room for improvement. Nevertheless, co-occurrence network analysis is based on statistical correlations and does not directly reflect true ecological interactions, nor should it be conflated with functional metabolic validation. These cross-kingdom findings should be interpreted as exploratory statistical signals rather than definitive biological interactions. Future functional validation (e.g., metatranscriptomics, metabolomics, in vitro co-culture) is required.

FUNGuild analysis provided exploratory evidence that fungal ecological guilds differed across cardiovascular health groups. The high-CVH group showed relatively greater representation of saprotrophic guilds related to plant residue or organic matter degradation, whereas the low-CVH group showed relatively higher representation of host-associated or potentially pathotrophic and symbiotrophic categories. This pattern may be consistent with the idea [47] that healthier dietary and metabolic environments are associated with fungal communities linked to plant-derived substrates, while less favorable host environments may be associated with shifts toward other ecological guilds. Although FUNGuild is an indirect ecological inference based on taxonomic annotation rather than a direct functional measurement, these patterns offer useful clues for future metagenomic and metabolomic studies of fungal contributions to metabolic health, bioactive compound processing, and physical function.

This study has several strengths. First, it extends a previously published bacterial microbiome analysis from the same TALENTs baseline by incorporating gut mycobiome profiling and bacterial–fungal cross-kingdom co-occurrence analysis, thereby providing a broader multi-kingdom view of microbiome patterns related to dietary inflammatory potential and multi-dimensional cardiovascular health in older adults. Second, the use of LE8_non-diet as a sensitivity outcome explicitly addressed the structural overlap between DII and the dietary component of LE8. Third, given the sparse and zero-inflated nature of fungal abundance data, we combined zero-inflation-aware approaches, Hurdle model sensitivity analysis, bootstrap resampling, and E-value assessment. This analytical strategy contributed to enhancing the credibility of the findings. Finally, the integration of diversity, co-occurrence networks, and FUNGuild-based functional predictions yields a prioritized set of testable hypotheses (five candidate genera and guild-level shifts) for future mechanistic studies. Although cross-sectional and exploratory, this work suggests that fungal and cross-kingdom features may provide additional ecological context beyond bacterial profiles alone, and offers a rationale for incorporating multi-kingdom measures in future nutritional and metabolic research.

Several limitations should be acknowledged. First, owing to the cross-sectional secondary design, the modest sample size, and the homogeneous age-restricted population, we cannot infer causality or temporality, and our results may not be generalizable to other populations. Second, dietary intake was evaluated with a 3-day dietary record without differentiation between weekdays and weekends. Despite training for participants to keep their usual diets, this method only captures short-term intake and may not adequately reflect long-term habitual dietary patterns. Furthermore, information on recent antibiotic and antifungal use, oral health status, and short-term dietary fungal exposure was lacking. Third, inherent limitations of ITS amplicon sequencing—including insufficient species-level taxonomic resolution, lack of direct information on fungal functional genes and metabolites, and the absence of DNA extraction and PCR negative controls in this study—restrict robust functional interpretation. The identification of key fungal genera and analyses of bacterial–fungal interactions presented herein are exploratory. Future studies integrating shotgun metagenomics, metatranscriptomics, metabolomics, inflammatory biomarkers, and experimental validation, preferably with longitudinal or interventional designs, are needed to verify and expand upon our observations.

## 5. Conclusions

In summary, this cross-sectional analysis characterizes gut mycobiome profiles and bacterial–fungal co-occurrence patterns in older adults stratified by dietary inflammatory potential and cardiovascular health status. At the community level, the most robust observation was that superior cardiovascular health was associated with higher fungal richness. Genus-level and cross-kingdom findings remain exploratory and cannot confirm causal mechanisms, yet they identify valuable candidate ecological signals and potential bacterial–fungal interactions. These results generate testable hypotheses for future nutritional and metabolic research incorporating lifestyle behaviors and functional health outcomes.

## Figures and Tables

**Table 1:** Demographic characteristics by DII group (*n* = 301).

Feature	LDII (*n* = 101)	MDII (*n* = 100)	HDII (*n* = 100)	*p*-Value
DII Score	−0.32 ± 0.86	1.29 ± 0.37	2.55 ± 0.43	<0.001 *
Age	65.58 ± 2.75	65.71 ± 2.60	65.69 ± 2.68	0.938
Body Mass Index (BMI)	24.66 ± 3.46	24.38 ± 3.22	24.61 ± 3.35	0.828
Gender Distribution	33 (32.7%)	29 (29.0%)	31 (31.0%)	0.853
Educational Level	28 (27.7%)	15 (15.0%)	18 (18.0%)	0.064
Residence Status	13 (12.9%)	10 (10.0%)	5 (5.0%)	0.151
Marital Status	80 (79.2%)	81 (81.0%)	87 (87.0%)	0.332
Income Level	29 (28.7%)	17 (17.0%)	13 (13.0%)	0.014 *
Smoking History	9 (8.9%)	7 (7.0%)	11 (11.0%)	0.613
Alcohol Consumption History	14 (13.9%)	15 (15.0%)	12 (12.0%)	0.823
Hypertension History	21 (20.8%)	24 (24.0%)	24 (24.0%)	0.822
Dyslipidemia History	8 (7.9%)	9 (9.0%)	14 (14.0%)	0.319
Diabetes History	11 (10.9%)	15 (15.0%)	16 (16.0%)	0.541

Continuous variables are expressed as mean ± SD, and categorical variables as n (%). Kruskal–Wallis H test was used for non-normally distributed DII scores; one-way ANOVA for other continuous variables, and χ^2^ test for categorical variables.* *p* < 0.05.

## Data Availability

The original contributions presented in the study are included in the article/Supplementary Material. Due to specific legal restrictions, the data are not publicly available, and further inquiries can be directed to the corresponding author.

## Supplementary Materials

The following supporting information can be downloaded at: https://www.mdpi.com/article/10.3390/metabo16080578/s1, Table S1: Scoring criteria for each component of Life’s Essential 8 (LE8); Table S2: Sensitivity analysis of DII with LE8_non-diet; Table S3: Fungal diversity and phylum-level differences across DII and CVH groups; Table S4: ZINB-derived fungal genera associated with DII and LE8; Table S5: Full bacterial–fungal cross-kingdom associations; Table S6: FUNGuild-based fungal ecological guilds across DII and CVH groups.
