# Supplementary material for: Dietary Inflammation, Gut Mycobiota, and Microbial Cross-Kingdom Associations with Cardiovascular Health in Older Adults
Source: Metabolites. 2026 Aug 16;16(8):578. doi: 10.3390/metabo16080578 (PMC13515542; doi:10.3390/metabo16080578)
Supplement: Supplementary file 1 [file metabolites-16-00578-s001.zip › metabolites-4419836-supplementary.pdf]

Supplementary materials

Supplementary Table S1. Scoring criteria for each component of Life's Essential 8 (LE8) 1
Supplementary Table S2. Sensitivity analysis of DII with LE8_non-diet 4
Supplementary Table S3. Fungal diversity and phylum-level differences across DII and CVH groups 5
Table S3A. Across DII groups 5
Table S3B. Across CVH groups 6
Supplementary Table S4. ZINB-derived fungal genera associated with DII and LE8 8
Table S4A. Fungal genera associated with DII in ZINB models 8
Table S4B. Fungal genera associated with total LE8 in ZINB models 10
Table S4C. Overlapping fungal genera associated with both DII and LE8 in ZINB models 14
Table S4D. Sensitivity analysis using Hurdle models 14
Table S4E. Bootstrap stability analysis for the 15 candidate fungal genera identified by ZINB models 15
Table S4F. E-value stability analysis for the 4 candidate fungal genera identified 16
Supplementary Table S5. Full bacterial–fungal cross-kingdom associations 16
Table S5A. Full bacterial–fungal cross-kingdom associations by ZINB 17
Table S5B. Significant bacterial–fungal associations identified based on DIABLO-selected features. 46
Supplementary Table S6. FUNGuild-based fungal ecological guilds across DII and CVH groups 49

Supplementary Table S1. Scoring criteria for each component of Life's Essential 8 (LE8)

Component	Criteria / Condition	Score (points)

Component	Criteria / Condition	Score (points)
Dietary score	MEPA score 15–16	100
	12–14	80
	8–11	50
	4–7	25
	0–3	0
Physical activity	Moderate-to-vigorous activity ≥150 min/week	100
	120–149 min/week	90
	90–119 min/week	80
	60–89 min/week	60
	1–29 min/week	20
	0 min/week	0
Nicotine exposure	Never smoked	100
	Quit >5 years ago	75
	Quit 1–5 years ago	50
	Quit <1 year ago or current e-cigarette use	25
	Current smoking	0
Sleep health	Average sleep duration 7–9 h/night	100
	>9–10 h	90
	6–7 h	70
	5–6 h or >10 h	40
	4–5 h	20
	<4 h	0
Body mass index (BMI)	<25.0 kg/m²	100
	25.0–29.9 kg/m²	70
	30.0–34.9 kg/m²	30
	35.0–39.9 kg/m²	15
	≥40.0 kg/m²	0
Blood pressure	<120 / <80 mmHg (normal)	100

Component	Criteria / Condition	Score (points)
	120–129 / <80 mmHg (elevated)	75
	130–139 / 80–89 mmHg (stage 1 hypertension)	50
	140–159 / 90–99 mmHg	25
	≥160 / ≥100 mmHg	0
Non-HDL cholesterol	<130 mg/dL	100
	130–159 mg/dL	60
	160–189 mg/dL	40
	190–219 mg/dL	20
	≥220 mg/dL	0
Blood glucose	No diabetes: fasting glucose ≤100 mg/dL or HbA1c <5.7%	100
	Prediabetes: fasting glucose 100–125 mg/dL or HbA1c 5.7%–6.4%	60
	Diabetes: HbA1c <7.0%	40
	HbA1c 7.0%–7.9%	30
	HbA1c 8.0%–8.9%	20
	HbA1c 9.0%–9.9%	10
	HbA1c ≥10.0%	0

Supplementary Table S2. Sensitivity analysis of DII with LE8_non-diet

DII	Outcome indicators	β(95%CI)[a]	*P*-value	β(95%CI)[b]	*P*-value
DII as continuous variable	LE8_non-diet	-1.106 (-2.158 to -0.054)	0.039*	-1.018 (-2.144 to 0.108)	0.0763
	total LE8 score[C]	-1.415(-2.346 to -0.483)	0.003**	-1.479(-2.478 to -0.479)	0.0039**
DII as categorical variable					
LDII	LE8_non-diet	Reference		Reference	
	Total LE8 score[C]	Reference		Reference	
MDII	LE8_non-diet	-1.535 (-4.924 to 1.855)	0.3737	-1.693 (-4.802to1.417)	0.2848
	Total LE8 score[C]	-2.067(-5.067 to 0.934)	0.1763	-2.249 (-5.014to 0.516)	0.1104
HDII	LE8_non-diet	-3.787 (-7.194 to -0.380)	0.029*	-2.573 (-6.019to0.873)	0.1427
	Total LE8 score[C]	-4.714(-7.729 to -1.698)	0.0023**	-3.880 (-6.945to -0.816)	0.0133*
Spearman correlation	LE8_non-diet	r = -0.130,p = 0.024*			
	Total LE8 score[C]	r = -0.17,p = 0.002**			

a Model 1: Unadjusted linear regression.

b Model 2: Adjusted for age, sex and energy intake, educational level, living situation, marital status, income level, BMI, smoking, alcohol consumption, digestive system diseases, and constipation presence.

c The results of the Total LE8 score have been previously reported

β values represent regression coefficients with 95% confidence intervals (CIs).

* *P* <0.05;** *P* <0.01; *** *P* <0.001.

Supplementary Table S3. Fungal diversity and phylum-level differences across DII and CVH groups

Table S3A. Across DII groups

Category	Specific metric	LDII(n=97)	MDII(n=96)	HDII(n=92)	Statistic/*P*-value
Alpha diversitya[a]	Chao1	59(37–87.75)	66.5(43–86)	60 (44.75–84)	0.649
	ACE	67 (43–92)	68 (46–89)	62 (51–89)	0.873
	Shannon	2.37(1.61–2.9)	2.51 (1.75–3.12)	2.4 (1.8–3.2)	0.51
Beta diversity[b]	weighted UniFrac distance	—	—	—	R^2=0.0107,*p*=0.197
	UnWeighted UniFrac distance	—	—	—	R^2=0.0088,*p*=0.488
Phylum-level relative abundance of fungi[c]	Ascomycota	84.41±22.03	82.31±21.47	84.71±18.8	0.665
	Basidiomycota	15.16±22.03	17.39±21.4	15.03±18.8	0.628
	Mucoromycota	0.05±0.19	0.08±0.4	0.05±0.22	0.709
	Rozellomycota	0.18±1.09	0.11±0.68	0.1±0.58	0.794
	Kickxellomycota	0.07±0.73	0±0	0±0.01	0.595
	Mortierellomycota	0.05±0.21	0.09±0.45	0.05±0.23	0.936
	Chytridiomycota	0.06±0.39	0.02±0.12	0.02±0.15	0.898
	Glomeromycota	0.01±0.05	0.02±0.12	0.02±0.19	0.551
	Entorrhizomycota	0±0	0±0	0±0.03	0.344
	Blastocladiomycota	0±0.03	0±0	0±0.02	0.595
	Zoopagomycota	0±0.01	0±0	0±0	0.143
	Calcarisporiellomycota	0±0.01	0±0	0±0	0.379
	Basidiobolomycota	0±0	0±0.01	0±0	0.379

a Values for fungal alpha diversity indices are presented as median (interquartile range, IQR); between-group differences were tested using the Kruskal–Wallis non-parametric test.

b Overall differences in fungal community structure (beta diversity) were assessed via permutational multivariate analysis of variance (PERMANOVA), with

R^2representing the proportion of variance explained by group.

c Values for fungal phylum-level relative abundance are presented as mean ± standard deviation (SD); between-group differences were tested using the Kruskal–Wallis non-parametric test.

* *P* <0.05;** *P* <0.01; *** *P* <0.001.

Table S3B. Across CVH groups

Category	Specific metric	LCVH (n=24)	MCVH (n=235)	HCVH(n=26)	Statistic/*P*-value
Alpha diversity[c]	Chao1	48.5(30.75–87.25)	59(41–84)	82(58.5–104.75)	0.018*
	ACE	52(31–88)	65(45–88)	86(62.25–107)	0.024*
	Shannon	2.22(1.58–2.73)	2.5(1.65–3.06)	2.4(1.77–3.29)	0.512
Beta diversity[d]	Weighted UniFrac distance	—	—	—	R^2=0.0087,*p*=0.447
	Unweighted UniFrac distance	—	—	—	R^2=0.0102,*p*=0.062
Phylum-level relative abundance of fungi[e]	Ascomycota	89.08±13.85	84.08±20.95	76.31±23.42	0.039*
	Basidiomycota	10.44±13.73	15.61±20.94	23.32±23.38	0.031*
	Mucoromycota	0.13±0.44	0.06±0.29	0.02±0.04	0.899
	Rozellomycota	0.19±0.93	0.13±0.83	0.12±0.6	0.463
	Kickxellomycota	0±0	0.03±0.47	0±0.01	0.131
	Mortierellomycota	0.04±0.19	0.06±0.3	0.16±0.46	0.3
	Chytridiomycota	0.05±0.25	0.03±0.26	0.03±0.13	0.557
	Glomeromycota	0.07±0.36	0.01±0.08	0.01±0.07	0.969
	Entorrhizomycota	0±0	0±0	0.01±0.05	0.007**
	Blastocladiomycota	0±0	0±0.02	0.01±0.03	0.131
	Zoopagomycota	0±0	0±0.01	0±0	0.808

Category	Specific metric	LCVH (n=24)	MCVH (n=235)	HCVH(n=26)	Statistic/*P*-value
	Calcarisporiellomycota	0±0	0±0	0±0.02	0.007**
	Basidiobolomycota	0±0	0±0.01	0±0	0.899

a Values for fungal alpha diversity indices are presented as median (interquartile range, IQR); between-group differences were tested using the Kruskal–Wallis non-parametric test.

b Overall differences in fungal community structure (beta diversity) were assessed via permutational multivariate analysis of variance (PERMANOVA), with R^2representing the proportion of variance explained by group.

c Values for fungal phylum-level relative abundance are presented as mean ± standard deviation (SD); between-group differences were tested using the Kruskal–Wallis non-parametric test.

* $P<0.05$;** $P<0.01$; *** $P<0.001$.

Supplementary Table S4. ZINB-derived fungal genera associated with DII and LE8

Table S4A. Fungal genera associated with DII in ZINB models

Genus	DII Estimate	DII *P*-value	DII Q-value	Prevalence(%)	Mean Relative Abundance(%)
Scopuloides	-41.046	<0.001	0.000	0.350	0.000
Cymostachys	242.529	<0.001	0.000	0.350	0.000
Sporothrix	-23.538	<0.001	0.000	0.700	0.013
Pseudothyridariella	-18.873	<0.001	0.000	0.700	0.006
Pleotrichocladium	0.818	<0.001	0.000	0.700	0.007
Inopinatum	1.196	<0.001	0.000	0.700	0.004
Caespitomonium	-1.261	<0.001	0.000	0.700	0.004
Cryptomarasmius	0.786	<0.001	0.000	0.700	0.004
Yamadazyma	0.953	<0.001	0.000	0.700	0.003
Roseodiscus	130.291	<0.001	0.000	0.350	0.000
Hypomyces	115.617	<0.001	0.000	0.350	0.000
Stachybotrys	0.565	<0.001	0.000	1.750	0.002
Fusicolla	-0.9	<0.001	0.000	0.700	0.000
Simplicillium	0.455	<0.001	0.000	0.700	0.001
Agrocybe	0.877	<0.001	0.000	0.700	0.000
Malassezia	0.615	<0.001	0.000	48.770	0.217
Trichaleurina	3.689	<0.001	0.000	0.700	0.000
Fusarium	-0.601	<0.001	0.000	31.230	1.356
Leotiomycetes_gen_Incertae_sedis	0.661	<0.001	0.000	1.750	0.003
Starmerella	3.717	<0.001	0.000	2.810	0.044
Xerochrysium	1.614	<0.001	0.001	2.460	0.010
Millerozyma	-1.934	<0.001	0.001	7.370	0.312
Kurtzmaniella	-0.442	<0.001	0.001	22.810	0.199

Genus	DII Estimate	DII *P*-value	DII Q-value	Prevalence(%)	Mean Relative Abundance(%)
Gomphillus	-0.456	<0.001	0.001	1.050	0.000
Hericium	1.628	<0.001	0.002	1.400	0.215
Aspergillaceae_gen_Incertae_sedis	-0.586	<0.001	0.004	31.230	0.460
Cyberlindnera	0.592	0.001	0.009	15.790	0.328
Paramyrothecium	0.914	0.001	0.009	2.110	0.016
Cryptococcus	0.744	0.001	0.010	7.370	0.055
Nectriaceae_gen_Incertae_sedis	-0.429	0.001	0.011	12.280	0.082
Cladosporium	-0.575	0.002	0.012	20.350	0.321
Tausonia	0.674	0.002	0.013	13.680	0.192
Paraphaeosphaeria	-0.747	0.002	0.014	2.810	0.034
Debaryomyces	-0.53	0.002	0.016	40.000	1.380
Agaricales_gen_Incertae_sedis	-0.517	0.003	0.022	11.580	0.089
Cutaneotrichosporon	1.074	0.004	0.027	9.820	0.276
Aspergillus	0.172	0.004	0.027	97.890	8.723
Saccharomycetales_gen_Incertae_sedis	0.467	0.006	0.035	35.790	0.725
Occultifur	0.304	0.007	0.039	8.770	0.051

Q-value is the *p*-value corrected for FDR.

Table S4B. Fungal genera associated with total LE8 in ZINB models

Genus	LE8 Estimate	LE8 *P*-value	LE8 FDR-corrected Q-value	Prevalence (%)	Mean Relative Abundance (%)
Pseudotremella	-11.682	0.000	0.000	0.350	0.002
Colacogloea	-12.087	0.000	0.000	0.350	0.002
Musicillium	-22.887	0.000	0.000	0.350	0.002
Lulworthiaceae_gen_Incertae_sedis	-15.398	0.000	0.000	0.350	0.002
Archaeorhizomycetales_gen_Incertae_sedis	4.369	0.000	0.000	0.350	0.001
Trametes	-6.539	0.000	0.000	0.350	0.001
Sporormiella	-6.824	0.000	0.000	0.350	0.001
Armillaria	-14.213	0.000	0.000	0.350	0.001
Cistella	-8.839	0.000	0.000	0.350	0.000
Gregarithecium	-13.581	0.000	0.000	0.350	0.000
Ripartites	-11.371	0.000	0.000	0.350	0.000
Pseudophialocephala	-7.710	0.000	0.000	0.350	0.000
Hypomyces	11.646	0.000	0.000	0.350	0.000
Parapyrenochaeta	-10.079	0.000	0.000	0.350	0.000
Scleroderma	2.882	0.000	0.000	0.350	0.000
Pseudorobillarda	10.218	0.000	0.000	0.350	0.000
Neofusicoccum	-9.204	0.000	0.000	0.350	0.000
Myxotrichaceae_gen_Incertae_sedis	5.389	0.000	0.000	0.350	0.000
Arrhenia	7.293	0.000	0.000	0.350	0.000
Valsaria	5.063	0.000	0.000	0.700	0.000
Leucosporidium	-11.137	0.000	0.000	0.350	0.002
Pleotrichocladium	-1.024	0.000	0.000	0.700	0.007
Sporopachydermia	-0.319	0.000	0.000	0.700	0.002

Genus	LE8 Estimate	LE8 *P*-value	LE8 FDR-corrected Q-value	Prevalence (%)	Mean Relative Abundance (%)
Inopinatum	1.914	0.000	0.000	0.700	0.004
Caespitomonium	-0.508	0.000	0.000	0.700	0.004
Lepidosphaeria	-10.140	0.000	0.000	0.350	0.000
Yamadazyma	-0.331	0.000	0.000	0.700	0.003
Magnibotryascoma	0.767	0.000	0.000	1.050	0.021
Vanrija	-0.134	0.000	0.000	0.700	0.001
Lophiostomataceae_gen_Incertae_sedis	-1.150	0.000	0.000	0.700	0.001
Sarcopodium	-0.098	0.000	0.000	0.700	0.002
Entyloma	0.070	0.000	0.000	1.050	0.002
Rhizoplaca	0.101	0.000	0.000	0.700	0.001
Diaporthe	-0.265	0.000	0.000	1.400	0.003
Phlebia	-0.339	0.000	0.000	0.700	0.003
Galerina	0.184	0.000	0.000	0.700	0.002
Lachancea	0.136	0.000	0.000	0.700	0.001
Septobasidiaceae_gen_Incertae_sedis	0.042	0.000	0.000	0.700	0.002
Lobulomycetales_gen_Incertae_sedis	18.329	0.000	0.000	0.350	0.000
Teunia	16.940	0.000	0.000	0.350	0.000
Achroiostachys	0.150	0.000	0.000	0.700	0.001
Simplicillium	0.163	0.000	0.000	0.700	0.001
Beauveria	17.081	0.000	0.000	0.350	0.000
Keissleriella	-0.068	0.000	0.000	1.750	0.003
Syncephalis	-0.835	0.000	0.000	0.700	0.000
Agrocybe	-0.346	0.000	0.000	0.700	0.000
Rhopalophora	0.044	0.000	0.000	0.700	0.002
Pithomyces	17.582	0.000	0.000	0.350	0.000
Trichaleurina	-0.633	0.000	0.000	0.700	0.000

Genus	LE8 Estimate	LE8 *P*-value	LE8 FDR-corrected Q-value	Prevalence (%)	Mean Relative Abundance (%)
Acremoniopsis	-0.060	0.000	0.000	0.700	0.000
Phoma	6.743	0.000	0.000	0.350	0.000
Claviceps	-0.018	0.000	0.000	0.700	0.001
Tilachlidium	0.051	0.000	0.000	0.700	0.001
Starmerella	0.179	0.000	0.001	2.810	0.044
Panaeolus	0.057	0.000	0.001	1.050	0.002
Pseudovalsaria	16.753	0.000	0.001	0.350	0.000
Dothidea	-0.079	0.000	0.001	1.050	0.000
Cyberlindnera	0.083	0.000	0.001	15.790	0.328
Cymostachys	17.180	0.000	0.001	0.350	0.000
Aspergillaceae_gen_Incertae_sedis	-0.079	0.000	0.002	31.230	0.460
Dioszegia	0.161	0.000	0.002	1.400	0.003
Tilletia	0.112	0.001	0.003	2.110	0.014
Geotrichum	-0.062	0.001	0.005	24.560	0.468
Dichotomopilus	-0.035	0.001	0.006	0.700	0.001
Saccharomycetales_gen_Incertae_sedis	-0.041	0.001	0.008	35.790	0.725
Biappendiculispora	15.521	0.002	0.008	0.350	0.000
Omphalina	15.521	0.002	0.008	0.350	0.000
Kurtzmaniella	0.071	0.003	0.012	22.810	0.199
Neocucurbitaria	-0.083	0.003	0.015	3.860	0.014
Papiliotrema	0.087	0.004	0.018	1.750	0.002
Aplosporella	-0.145	0.004	0.019	1.750	0.057
Actinomucor	-0.230	0.005	0.022	0.700	0.000
Nigrospora	-0.051	0.006	0.026	18.250	0.281
Xerochrysium	-0.095	0.007	0.030	2.460	0.010
Cercospora	0.082	0.007	0.031	2.110	0.009

Genus	LE8 Estimate	LE8 *P*-value	LE8 FDR-corrected Q-value	Prevalence (%)	Mean Relative Abundance (%)
Entoloma	0.051	0.008	0.032	1.750	0.003
Saturnispora	-0.054	0.008	0.032	1.050	0.000
Sakaguchia	0.054	0.010	0.042	1.050	0.000

R-value is the *p*-value corrected for FDR.

Table S4C. Overlapping fungal genera associated with both DII and LE8 in ZINB models

Genus	DII Estimate	DII *P*-value	DII Q-value	LE8 Estimate	LE8 *P-value*	LE8 Q-value	Direction Pattern	Opposite Direction between DII and LE8
Pleotrichocladium	0.818	<0.001	0	-1.024	<0.001	0	DII↑, LE8↓	Yes
Yamadazyma	0.953	<0.001	0	-0.331	<0.001	0	DII↑, LE8↓	Yes
Agrocybe	0.877	<0.001	0	-0.346	<0.001	0	DII↑, LE8↓	Yes
Trichaleurina	3.689	<0.001	0	-0.633	<0.001	0	DII↑, LE8↓	Yes
Xerochrysium	1.614	<0.001	0.001	-0.095	0.007	0.03	DII↑, LE8↓	Yes
Kurtzmaniella	-0.442	<0.001	0.001	0.07	0.003	0.012	DII↓, LE8↑	Yes
Saccharomycetales_gen_Incertae_sedis	0.467	0.006	0.035	-0.041	0.001	0.008	DII↑, LE8↓	Yes
Cymostachys	242.529	<0.001	0	17.18	<0.001	0.001	DII↑, LE8↑	No
Inopinatum	1.196	<0.001	0	1.914	<0.001	0	DII↑, LE8↑	No
Caespitomonium	-1.261	<0.001	0	-0.508	<0.001	0	DII↓, LE8↓	No
Hypomyces	115.617	<0.001	0	11.646	<0.001	0	DII↑, LE8↑	No
Simplicillium	0.455	<0.001	0	0.163	<0.001	0	DII↑, LE8↑	No
Starmerella	3.717	<0.001	0	0.179	<0.001	0.001	DII↑, LE8↑	No
Aspergillaceae_gen_Incertae_sedis	-0.586	<0.001	0.004	-0.079	<0.001	0.002	DII↓, LE8↓	No
Cyberlindnera	0.592	0.001	0.009	0.083	<0.001	0.001	DII↑, LE8↑	No

S-value is the *p*-value corrected for FDR.

Table S4D. Sensitivity analysis using Hurdle models

**Genus**	**DII Count_ Estimate**	**DII Count_ FDR**	**DII Zero_ Estimate**	**DII Zero_ FDR**	**LE8 Count_ Estimate**	**LE8 Count_ FDR**	**LE8 Zero_ Estimate**	**LE8 Zero_ FDR**	**ZINB Consistency DII/LE8**

Genus	DII Count_ Estimate	DII Count_ FDR	DII Zero_ Estimate	DII Zero_ FDR	LE8 Count_ Estimate	LE8 Count_ FDR	LE8 Zero_ Estimate	LE8 Zero_ FDR	ZINB Consistency DII/LE8
Inopinatum	1.195	0	-0.162	0.971	1.881	0	-0.059	0.842	Yes/ Yes
Yamadazyma	0.953	0	-0.469	0.928	-0.328	0	-0.123	0.803	Yes/ Yes
Simplicillium	0.455	0	0.369	0.961	0.196	1	0.147	0.803	Yes/ No
Kurtzmaniella	-0.442	0	0.062	0.961	0.069	0.023	-0.011	0.856	Yes/ Yes
Starmerella	3.285	0.001	-0.055	0.972	0.186	0.254	0.022	0.892	Yes/ No
Aspergillaceae_gen_Incertae_sedis	-0.59	0.002	0.012	0.976	-0.078	0.003	0.018	0.803	Yes/ Yes
Cyberlindnera	0.596	0.004	-0.025	0.971	0.083	0.001	0.021	0.803	Yes/ Yes
Saccharomycetales_gen_Incertae_sedis	0.468	0.019	0.118	0.919	-0.041	0.014	0.004	0.914	Yes/ Yes
Xerochrysium	23.079	NA	-0.722925326	0.919355409	-0.121	1	0.043	0.837	NA/ NA
Pleotrichocladium	57.426	NA	-0.184816759	0.970994934	-0.577	1	-0.019	0.921	NA/ NA
Caespitomonium	NA	NA	NA	NA	-0.436	1	0.002	0.982	NA/ NA
Agrocybe	NA	NA	NA	NA	-0.413	1	0.004	0.972	NA/ NA
Trichaleurina	NA	NA	NA	NA	-0.776	NA	-0.003	0.980	NA/ NA
Hypomyces	-3.242	NA	-1.096849115	0.919355409	-0.191	NA	-0.094	0.837	NA/ NA
Cymostachys	2.751	NA	-0.071585689	0.976052841	0.031	NA	-0.063	0.892	NA/ NA

Table S4E. Bootstrap stability analysis for the 15 candidate fungal genera identified by ZINB models

Genus	DII_Stability	LE8_Stability	DII_Converged_Reps	LE8_Converged_Reps	DII_Stable	LE8_Stable
Cymostachys	0.069	0.207	619	630	No	No
Pleotrichocladium	0.181	0.304	786	843	No	No
Inopinatum	0.264	0.306	797	843	No	No
Caespitomonium	0.166	0.332	612	830	No	No
Yamadazyma	0.318	0.419	692	859	No	No

Hypomyces	0.081	0.18	250	549	No	No
Simplicillium	0.186	0.442	676	859	No	No
Agrocybe	0.224	0.291	689	759	No	No
Trichaleurina	0.169	0.454	566	852	No	No
Starmerella	0.501	0.511	666	963	Yes	Yes
Xerochrysium	0.395	0.606	691	996	No	Yes
Kurtzmaniella	0.731	0.666	995	998	Yes	Yes
Aspergillaceae_gen_Incertae_sedis	0.68	0.792	994	998	Yes	Yes
Cyberlindnera	0.784	0.833	975	1000	Yes	Yes
Saccharomycetales_gen_Incertae_sedis	0.715	0.747	993	990	Yes	Yes

Bootstrap = 1000 repetitions

Table S4F. E-value stability analysis for the 4 candidate fungal genera identified

Genus	LE8_Estimate	LE8_ RR	LE8_ Evalue	LE8_Evalue_ LCI	DII_Estimate	DII_ RR	DII_ Evalue	DII_Evalue_ LCI
Kurtzmaniella	0.070499238	1.073	1.35	1.19	-0.442064812	0.643	2.49	3.29
Aspergillaceae_gen_Incertae_sedis	-0.07871305	0.924	1.38	1.51	-0.58624967	0.556	2.99	4.43
Cyberlindnera	0.083216257	1.087	1.39	1.25	0.592135173	1.808	3.02	1.86
Saccharomycetales_gen_Incertae_sedis	-0.041432135	0.959	1.25	1.34	0.467194746	1.596	2.57	1.55

Supplementary Table S5. Full bacterial–fungal cross-kingdom associations

Table S5A. Full bacterial–fungal cross-kingdom associations by ZINB

Bacterial_Genus	Fungal_Genus	Association_Estimate	Standard_Error	P_value	Q_value	Association_Direction
Pseudobutyrivibrio	Millerozyma	4.4221	1.3119	0.0007	0.0152	Positive
Odoribacter	Kazachstania	2.0001	0.5490	0.0003	0.0076	Positive
Butyricicoccus	Wickerhamiella	1.8773	0.5737	0.0011	0.0191	Positive
Odoribacter	Kodamaea	1.7766	0.5082	0.0005	0.0109	Positive
Butyricimonas	Acremonium	1.2566	0.2858	0.0000	0.0008	Positive
Parasutterella	Cryptococcus	1.2087	0.2266	0.0000	0.0000	Positive
Parasutterella	Millerozyma	1.0487	0.2995	0.0005	0.0109	Positive
Propionibacterium	Kurtzmaniella	0.9430	0.2935	0.0013	0.0223	Positive
Butyricicoccus	Debaryomyces	0.8013	0.1995	0.0001	0.0027	Positive
Bilophila	Acremonium	0.7008	0.2107	0.0009	0.0167	Positive

Bacterial_Genus	Fungal_Genus	Association_Estimate	Standard_Error	P_value	Q_value	Association_Direction
Faecalicoccus	Wallemia	0.5658	0.1851	0.0022	0.0317	Positive
Sutterella	Kodamaea	0.5190	0.1446	0.0003	0.0086	Positive
Sutterella	Coprinopsis	0.4983	0.1647	0.0025	0.0339	Positive
Clostridium_XlVb	Debaryomyces	0.4814	0.1234	0.0001	0.0040	Positive
Faecalicoccus	Cladosporium	0.4800	0.1513	0.0015	0.0241	Positive
Parasutterella	Eremothecium	0.3502	0.1174	0.0029	0.0372	Positive
Paraprevotella	Debaryomyces	0.2954	0.0819	0.0003	0.0084	Positive
Clostridium_XlVb	Cyberlindnera	0.2809	0.0976	0.0040	0.0475	Positive
Paraprevotella	Acremonium	0.2648	0.0560	0.0000	0.0003	Positive
Parabacteroides	Acremonium	0.2054	0.0616	0.0009	0.0165	Positive
Coprococcus	Acremonium	0.1448	0.0502	0.0039	0.0466	Positive
Dialister	Aspergillaceae_gen_Incertae_sedis	0.1444	0.0273	0.0000	0.0000	Positive
Blautia	Phomatospora	0.1129	0.0294	0.0001	0.0045	Positive
Ruminococcus	Debaryomyces	0.0888	0.0247	0.0003	0.0086	Positive
Coprococcus	Fungi_gen_Incertae_sedis	0.0844	0.0288	0.0033	0.0418	Positive

Bacterial_Genus	Fungal_Genus	Association_Estimate	Standard_Error	P_value	Q_value	Association_Direction
Phascolarctobacterium	Erythrobasidium	0.0765	0.0228	0.0008	0.0159	Positive
Ruminococcus	Acremonium	0.0671	0.0195	0.0006	0.0125	Positive
Lachnospiracea_incertae_sedis	Kurtzmaniella	0.0640	0.0161	0.0001	0.0030	Positive
Blautia	Didymosphaeriaceae_gen_Incertae_sedis	0.0632	0.0201	0.0017	0.0263	Positive
Lachnospiracea_incertae_sedis	Nakaseomyces	0.0596	0.0154	0.0001	0.0042	Positive
Lachnospiracea_incertae_sedis	Debaryomyces	0.0553	0.0093	0.0000	0.0000	Positive
Clostridium_XlVa	Debaryomyces	0.0496	0.0106	0.0000	0.0003	Positive
Lachnospiracea_incertae_sedis	Exophiala	0.0349	0.0110	0.0015	0.0241	Positive
Phascolarctobacterium	Acremonium	0.0319	0.0098	0.0011	0.0199	Positive
Phascolarctobacterium	Ascomycota_gen_Incertae_sedis	0.0263	0.0088	0.0027	0.0365	Positive
Faecalibacterium	Agaricales_gen_Incertae_sedis	0.0209	0.0056	0.0002	0.0055	Positive
Megamonas	Millerozyma	0.0202	0.0050	0.0001	0.0027	Positive
Bacteroides	Wickerhamomyces	0.0114	0.0037	0.0019	0.0286	Positive

Bacterial_Genus	Fungal_Genus	Association_Estimate	Standard_Error	P_value	Q_value	Association_Direction
Prevotella	Aspergillaceae_gen_Incertae_sedis	0.0106	0.0016	0.0000	0.0000	Positive
Megamonas	Meyerozyma	0.0094	0.0028	0.0007	0.0152	Positive
Prevotella	Erythrobasidium	0.0078	0.0017	0.0000	0.0005	Positive
Prevotella	Kurtzmaniella	0.0070	0.0022	0.0017	0.0270	Positive
Megamonas	Aspergillus	-0.0053	0.0017	0.0023	0.0324	Negative
Prevotella	Dipodascaceae_gen_Incertae_sedis	-0.0057	0.0016	0.0003	0.0086	Negative
Prevotella	Tausonia	-0.0067	0.0020	0.0009	0.0165	Negative
Prevotella	Debaryomyces	-0.0075	0.0014	0.0000	0.0000	Negative
Prevotella	Wickerhamomyces	-0.0082	0.0022	0.0003	0.0076	Negative
Prevotella	Sterigmatomyces	-0.0094	0.0027	0.0005	0.0108	Negative
Bacteroides	Erythrobasidium	-0.0119	0.0036	0.0009	0.0167	Negative
Megamonas	Rozellomycota_gen_Incertae_sedis	-0.0124	0.0035	0.0004	0.0095	Negative
Faecalibacterium	Cystobasidium	-0.0130	0.0044	0.0035	0.0431	Negative
Megamonas	Dipodascaceae_gen_Incertae_sedis	-0.0134	0.0036	0.0002	0.0060	Negative

Bacterial_Genus	Fungal_Genus	Association_Estimate	Standard_Error	P_value	Q_value	Association_Direction
Prevotella	Rhizopus	-0.0140	0.0036	0.0001	0.0041	Negative
Megamonas	Acremonium	-0.0149	0.0042	0.0004	0.0090	Negative
Megasphaera	Debaryomyces	-0.0150	0.0044	0.0006	0.0133	Negative
Bacteroides	Aspergillaceae_gen_Incertae_sedis	-0.0153	0.0032	0.0000	0.0003	Negative
Klebsiella	Wallemia	-0.0163	0.0045	0.0003	0.0085	Negative
Klebsiella	Rhodotorula	-0.0166	0.0054	0.0020	0.0292	Negative
Faecalibacterium	Erythrobasidium	-0.0170	0.0056	0.0023	0.0326	Negative
Megamonas	Neosetophoma	-0.0175	0.0046	0.0001	0.0048	Negative
	Pleurotus	-0.0178	0.0048	0.0003	0.0073	Negative
Bifidobacterium	Wallemia	-0.0179	0.0054	0.0010	0.0182	Negative
Prevotella	Millerozyma	-0.0180	0.0058	0.0019	0.0286	Negative
Megasphaera	Wallemia	-0.0183	0.0040	0.0000	0.0004	Negative
Megasphaera	Cladosporium	-0.0184	0.0049	0.0002	0.0055	Negative
Megasphaera	Colletotrichum	-0.0200	0.0068	0.0034	0.0421	Negative
Bifidobacterium	Acremonium	-0.0203	0.0050	0.0000	0.0025	Negative

Bacterial_Genus	Fungal_Genus	Association_Estimate	Standard_Error	P_value	Q_value	Association_Direction
Megasphaera	Nectriaceae_gen_Incertae_sedis	-0.0209	0.0066	0.0016	0.0256	Negative
Faecalibacterium	Saccharomycetales_gen_Incertae_sedis	-0.0217	0.0054	0.0000	0.0025	Negative
Faecalibacterium	Nectriaceae_gen_Incertae_sedis	-0.0225	0.0072	0.0018	0.0273	Negative
Lachnospiracea_incertae_sedis	Dipodascaceae_gen_Incertae_sedis	-0.0230	0.0068	0.0007	0.0144	Negative
Klebsiella	Neovaginatispora	-0.0234	0.0053	0.0000	0.0008	Negative
Blautia	Wallemia	-0.0239	0.0084	0.0042	0.0488	Negative
Gemmiger	Sordariomycetes_gen_Incertae_sedis	-0.0251	0.0079	0.0015	0.0241	Negative
Bifidobacterium	Psathyrellaceae_gen_Incertae_sedis	-0.0260	0.0087	0.0029	0.0372	Negative
Roseburia	Meyerozyma	-0.0261	0.0084	0.0019	0.0286	Negative
Klebsiella	Aspergillaceae_gen_Incertae_sedis	-0.0279	0.0093	0.0028	0.0369	Negative
Klebsiella	Fusarium	-0.0284	0.0050	0.0000	0.0000	Negative
Klebsiella	Meyerozyma	-0.0298	0.0051	0.0000	0.0000	Negative
Megasphaera	Rhodotorula	-0.0301	0.0059	0.0000	0.0001	Negative

Bacterial_Genus	Fungal_Genus	Association_Estimate	Standard_Error	P_value	Q_value	Association_Direction
Klebsiella	Monascus	-0.0303	0.0066	0.0000	0.0004	Negative
Klebsiella	Colletotrichum	-0.0307	0.0060	0.0000	0.0001	Negative
Megamonas	Trichoderma	-0.0311	0.0108	0.0039	0.0464	Negative
Blautia	Meyerozyma	-0.0318	0.0104	0.0023	0.0322	Negative
Megasphaera	Kurtzmaniella	-0.0319	0.0100	0.0014	0.0231	Negative
Clostridium_XlVa	Meyerozyma	-0.0329	0.0107	0.0022	0.0312	Negative
	Millerozyma	-0.0346	0.0091	0.0001	0.0051	Negative
Roseburia	Cladosporium	-0.0348	0.0117	0.0029	0.0372	Negative
Megasphaera	Meyerozyma	-0.0354	0.0087	0.0000	0.0024	Negative
Acidaminococcus	Fungi_gen_Incertae_sedis	-0.0359	0.0096	0.0002	0.0055	Negative
Phascolarctobacterium	Wickerhamomyces	-0.0363	0.0081	0.0000	0.0005	Negative
Roseburia	Erythrobasidium	-0.0365	0.0102	0.0003	0.0086	Negative
Bifidobacterium	Sterigmatomyces	-0.0368	0.0098	0.0002	0.0053	Negative
	Phallus	-0.0368	0.0097	0.0001	0.0051	Negative
Klebsiella	Kazachstania	-0.0386	0.0076	0.0000	0.0001	Negative
Bifidobacterium	Montagnula	-0.0387	0.0106	0.0003	0.0073	Negative

Bacterial_Genus	Fungal_Genus	Association_Estimate	Standard_Error	P_value	Q_value	Association_Direction
Megamonas	Mucor	-0.0399	0.0118	0.0007	0.0149	Negative
Blautia	Aspergillaceae_gen_Incertae_sedis	-0.0413	0.0108	0.0001	0.0046	Negative
Oscillibacter	Didymocyrtis	-0.0420	0.0115	0.0003	0.0076	Negative
Megamonas	Nectriaceae_gen_Incertae_sedis	-0.0423	0.0123	0.0006	0.0128	Negative
Bifidobacterium	Geotrichum	-0.0423	0.0121	0.0005	0.0110	Negative
Lachnospiracea_incertae_sedis	Didymosphaeriaceae_gen_Incertae_sedis	-0.0426	0.0134	0.0015	0.0241	Negative
Bifidobacterium	Phaeosphaeria	-0.0431	0.0105	0.0000	0.0022	Negative
Prevotella	Phallus	-0.0443	0.0085	0.0000	0.0000	Negative
Escherichia	Sporidiobolales_gen_Incertae_sedis	-0.0454	0.0124	0.0003	0.0074	Negative
Clostridium_XlVa	Exophiala	-0.0458	0.0116	0.0001	0.0033	Negative
Roseburia	Kazachstania	-0.0460	0.0153	0.0027	0.0365	Negative
Phascolarctobacterium	Onygenales_gen_Incertae_sedis	-0.0462	0.0162	0.0042	0.0490	Negative
Gemmiger	Pleurotus	-0.0487	0.0136	0.0003	0.0086	Negative
Oscillibacter	Aspergillaceae_gen_Incertae_sedis	-0.0493	0.0161	0.0022	0.0317	Negative

Bacterial_Genus	Fungal_Genus	Association_Estimate	Standard_Error	P_value	Q_value	Association_Direction
Megasphaera	Mucor	-0.0496	0.0159	0.0018	0.0270	Negative
Klebsiella	Kurtzmaniella	-0.0507	0.0131	0.0001	0.0043	Negative
Roseburia	Aspergillaceae_gen_Incertae_sedis	-0.0553	0.0188	0.0033	0.0410	Negative
Oscillibacter	Exophiala	-0.0554	0.0160	0.0005	0.0118	Negative
Blautia	Sordariomycetes_gen_Incertae_sedis	-0.0566	0.0140	0.0001	0.0027	Negative
Ruminococcus2	Malassezia	-0.0575	0.0186	0.0020	0.0292	Negative
Gemmiger	Cryptococcus	-0.0617	0.0172	0.0003	0.0085	Negative
Megamonas	Eremothecium	-0.0619	0.0134	0.0000	0.0004	Negative
Gemmiger	Saccharomycopsis	-0.0622	0.0168	0.0002	0.0063	Negative
Ruminococcus2	Xerogeomyces	-0.0648	0.0207	0.0018	0.0271	Negative
Klebsiella	Pleurotus	-0.0654	0.0147	0.0000	0.0006	Negative
Eubacterium	Fusarium	-0.0656	0.0193	0.0007	0.0140	Negative
Ruminococcus2	Pichia	-0.0661	0.0207	0.0014	0.0235	Negative
Oscillibacter	Fusarium	-0.0661	0.0146	0.0000	0.0005	Negative

Bacterial_Genus	Fungal_Genus	Association_Estimate	Standard_Error	P_value	Q_value	Association_Direction
Ruminococcus	Erythrobasidium	-0.0664	0.0169	0.0001	0.0037	Negative
Klebsiella	Phallus	-0.0670	0.0223	0.0026	0.0358	Negative
Blautia	Erythrobasidium	-0.0677	0.0222	0.0023	0.0319	Negative
Alloprevotella	Zygosaccharomyces	-0.0685	0.0232	0.0031	0.0399	Negative
Paraprevotella	Saccharomyces	-0.0685	0.0237	0.0039	0.0463	Negative
Megasphaera	Kodamaea	-0.0700	0.0194	0.0003	0.0083	Negative
Roseburia	Saccharomycopsis	-0.0710	0.0201	0.0004	0.0100	Negative
Blautia	Pichia	-0.0732	0.0136	0.0000	0.0000	Negative
Alloprevotella	Xeromyces	-0.0733	0.0257	0.0043	0.0492	Negative
Roseburia	Cryptococcus	-0.0745	0.0234	0.0014	0.0237	Negative
Phascolarctobacterium	Cladosporium	-0.0764	0.0174	0.0000	0.0008	Negative
Fusobacterium	Meyerozyma	-0.0794	0.0238	0.0008	0.0162	Negative
Oscillibacter	Malassezia	-0.0810	0.0186	0.0000	0.0008	Negative
Alistipes	Psathyrellaceae_gen_Incertae_sedis	-0.0836	0.0275	0.0024	0.0329	Negative
Alistipes	Coprinellus	-0.0836	0.0244	0.0006	0.0133	Negative

Bacterial_Genus	Fungal_Genus	Association_Estimate	Standard_Error	P_value	Q_value	Association_Direction
Ruminococcus2	Aspergillaceae_gen_Incertae_sedis	-0.0845	0.0294	0.0041	0.0481	Negative
Dialister	Debaryomyces	-0.0845	0.0255	0.0009	0.0169	Negative
Ruminococcus2	Dipodascaceae_gen_Incertae_sedis	-0.0845	0.0206	0.0000	0.0022	Negative
Eubacterium	Sordariomycetes_gen_Incertae_sedis	-0.0855	0.0243	0.0004	0.0105	Negative
Clostridium_XlVa	Erythrobasidium	-0.0879	0.0167	0.0000	0.0000	Negative
Alistipes	Coprinopsis	-0.0900	0.0262	0.0006	0.0129	Negative
Streptococcus	Dipodascaceae_gen_Incertae_sedis	-0.0902	0.0301	0.0027	0.0365	Negative
Ruminococcus	Aspergillaceae_gen_Incertae_sedis	-0.0913	0.0199	0.0000	0.0004	Negative
Parabacteroides	Aspergillaceae_gen_Incertae_sedis	-0.0938	0.0303	0.0019	0.0287	Negative
Alistipes	Naganishia	-0.0941	0.0283	0.0009	0.0169	Negative
Barnesiella	Sordariomycetes_gen_Incertae_sedis	-0.0954	0.0295	0.0012	0.0207	Negative
Acetanaerobacterium	Vishniacozyma	-0.0973	0.0320	0.0024	0.0329	Negative
Alistipes	Erythrobasidium	-0.0978	0.0340	0.0040	0.0476	Negative

Bacterial_Genus	Fungal_Genus	Association_Estimate	Standard_Error	P_value	Q_value	Association_Direction
Anaerostipes	Wallemia	-0.0979	0.0312	0.0017	0.0265	Negative
Flavonifractor	Penicillium	-0.0983	0.0335	0.0034	0.0418	Negative
Alistipes	Exophiala	-0.1040	0.0255	0.0000	0.0024	Negative
Blautia	Cryptococcus	-0.1049	0.0255	0.0000	0.0021	Negative
Anaerostipes	Penicillium	-0.1064	0.0281	0.0002	0.0053	Negative
Fusobacterium	Geotrichum	-0.1073	0.0230	0.0000	0.0003	Negative
Lachnospira	Fusarium	-0.1117	0.0358	0.0018	0.0274	Negative
Dialister	Coprinopsis	-0.1150	0.0232	0.0000	0.0001	Negative
Alistipes	Talaromyces	-0.1199	0.0299	0.0001	0.0027	Negative
Roseburia	Rhizopus	-0.1212	0.0258	0.0000	0.0003	Negative
Oscillibacter	Rhizopus	-0.1227	0.0425	0.0039	0.0463	Negative
Ruminococcus	Geotrichum	-0.1242	0.0388	0.0014	0.0230	Negative
Clostridium_sensu_stricto	Candida	-0.1245	0.0357	0.0005	0.0111	Negative
Roseburia	Ganoderma	-0.1255	0.0269	0.0000	0.0003	Negative
Alistipes	Didymocyrtis	-0.1298	0.0378	0.0006	0.0128	Negative
Romboutsia	Penicillium	-0.1314	0.0450	0.0035	0.0431	Negative

Bacterial_Genus	Fungal_Genus	Association_Estimate	Standard_Error	P_value	Q_value	Association_Direction
Alistipes	Paecilomyces	-0.1339	0.0282	0.0000	0.0003	Negative
Gemmiger	Millerozyma	-0.1403	0.0369	0.0001	0.0051	Negative
Collinsella	Malassezia	-0.1430	0.0478	0.0028	0.0365	Negative
Raoultella	Malassezia	-0.1488	0.0519	0.0041	0.0481	Negative
Flavonifractor	Wickerhamomyces	-0.1522	0.0452	0.0007	0.0152	Negative
Megasphaera	Paecilomyces	-0.1522	0.0461	0.0010	0.0175	Negative
Alistipes	Cystobasidium	-0.1574	0.0515	0.0022	0.0317	Negative
Blautia	Kodamaea	-0.1612	0.0518	0.0018	0.0280	Negative
Flavonifractor	Pichia	-0.1620	0.0348	0.0000	0.0003	Negative
Barnesiella	Didymosphaeriaceae_gen_Incertae_sedis	-0.1635	0.0451	0.0003	0.0080	Negative
Ruminococcus	Phallus	-0.1677	0.0555	0.0025	0.0340	Negative
Acetanaerobacterium	Didymosphaeriaceae_gen_Incertae_sedis	-0.1689	0.0530	0.0014	0.0237	Negative
Alloprevotella	Hanseniaspora	-0.1689	0.0579	0.0035	0.0437	Negative
Alistipes	Nectriaceae_gen_Incertae_sedis	-0.1732	0.0535	0.0012	0.0208	Negative

Bacterial_Genus	Fungal_Genus	Association_Estimate	Standard_Error	P_value	Q_value	Association_Direction
Collinsella	Nectriaceae_gen_Incertae_sedis	-0.1755	0.0541	0.0012	0.0203	Negative
Collinsella	Nigrospora	-0.1791	0.0445	0.0001	0.0027	Negative
Romboutsia	Tausonia	-0.1793	0.0561	0.0014	0.0230	Negative
Acetanaerobacterium	Exophiala	-0.1801	0.0631	0.0043	0.0495	Negative
Escherichia	Neovaginatispora	-0.1895	0.0414	0.0000	0.0004	Negative
Eubacterium	Nectriaceae_gen_Incertae_sedis	-0.1930	0.0495	0.0001	0.0040	Negative
Barnesiella	Exophiala	-0.1938	0.0515	0.0002	0.0054	Negative
Desulfovibrio	Occultifur	-0.1990	0.0664	0.0027	0.0365	Negative
Flavonifractor	Kazachstania	-0.2025	0.0478	0.0000	0.0014	Negative
Acetanaerobacterium	Coprinopsis	-0.2027	0.0654	0.0019	0.0287	Negative
Alloprevotella	Xerogeomyces	-0.2089	0.0454	0.0000	0.0004	Negative
Eubacterium	Aspergillaceae_gen_Incertae_sedis	-0.2095	0.0376	0.0000	0.0000	Negative
Anaerostipes	Kazachstania	-0.2098	0.0556	0.0002	0.0053	Negative
Ruminococcus2	Naganishia	-0.2105	0.0497	0.0000	0.0014	Negative

Bacterial_Genus	Fungal_Genus	Association_Estimate	Standard_Error	P_value	Q_value	Association_Direction
Akkermansia	Nigrospora	-0.2130	0.0741	0.0041	0.0476	Negative
Coprococcus	Aspergillaceae_gen_Incertae_sedis	-0.2167	0.0660	0.0010	0.0184	Negative
Clostridium_IV	Deconica	-0.2190	0.0720	0.0024	0.0329	Negative
Collinsella	Pichia	-0.2214	0.0547	0.0001	0.0026	Negative
Barnesiella	Sebacina	-0.2238	0.0724	0.0020	0.0292	Negative
Clostridium_sensu_stricto	Wickerhamiella	-0.2251	0.0779	0.0039	0.0463	Negative
Lactobacillus	Ascomycota_gen_Incertae_sedis	-0.2253	0.0759	0.0030	0.0389	Negative
Clostridium_sensu_stricto	Deconica	-0.2255	0.0684	0.0010	0.0179	Negative
Phascolarctobacterium	Nakaseomyces	-0.2303	0.0746	0.0020	0.0294	Negative
Streptococcus	Pichia	-0.2311	0.0608	0.0001	0.0051	Negative
Lactobacillus	Nectriaceae_gen_Incertae_sedis	-0.2347	0.0813	0.0039	0.0463	Negative
Desulfovibrio	Dipodascaceae_gen_Incertae_sedis	-0.2351	0.0771	0.0023	0.0322	Negative
Collinsella	Aspergillaceae_gen_Incertae_sedis	-0.2352	0.0456	0.0000	0.0001	Negative
Blautia	Nectriaceae_gen_Incertae_s	-0.2392	0.0638	0.0002	0.0055	Negative

Bacterial_Genus	Fungal_Genus	Association_Estimate	Standard_Error	P_value	Q_value	Association_Direction
	edis					
Collinsella	Meyerozyma	-0.2427	0.0656	0.0002	0.0065	Negative
Barnesiella	Coprinellus	-0.2446	0.0806	0.0024	0.0329	Negative
Clostridium_sensu_stricto	Acremonium	-0.2467	0.0681	0.0003	0.0080	Negative
Romboutsia	Xeromyces	-0.2497	0.0745	0.0008	0.0159	Negative
Barnesiella	Dipodascaceae_gen_Incertae_sedis	-0.2549	0.0711	0.0003	0.0086	Negative
Lactobacillus	Nigrospora	-0.2565	0.0871	0.0032	0.0408	Negative
Eubacterium	Naganishia	-0.2590	0.0877	0.0031	0.0399	Negative
Parabacteroides	Naganishia	-0.2604	0.0887	0.0033	0.0418	Negative
Lachnospira	Coprinopsis	-0.2646	0.0633	0.0000	0.0016	Negative
Collinsella	Kurtzmaniella	-0.2754	0.0584	0.0000	0.0003	Negative
Collinsella	Xeromyces	-0.2780	0.0887	0.0017	0.0267	Negative
Collinsella	Cyberlindnera	-0.2809	0.0607	0.0000	0.0004	Negative
Clostridium_sensu_stricto	Aspergillaceae_gen_Incertae_sedis	-0.2862	0.0806	0.0004	0.0095	Negative

Bacterial_Genus	Fungal_Genus	Association_Estimate	Standard_Error	P_value	Q_value	Association_Direction
Butyricicoccus	Periconia	-0.2931	0.0994	0.0032	0.0406	Negative
Desulfovibrio	Sampaiozyma	-0.2957	0.1034	0.0042	0.0490	Negative
Clostridium_XVIII	Kazachstania	-0.2966	0.1024	0.0038	0.0456	Negative
Barnesiella	Erythrobasidium	-0.2974	0.0819	0.0003	0.0080	Negative
Ruminococcus2	Ganoderma	-0.3013	0.0975	0.0020	0.0292	Negative
Flavonifractor	Erythrobasidium	-0.3035	0.0721	0.0000	0.0015	Negative
Desulfovibrio	Basidiomycota_gen_Incertae_sedis	-0.3045	0.0914	0.0009	0.0165	Negative
Dorea	Exophiala	-0.3095	0.1037	0.0028	0.0372	Negative
Dorea	Aspergillaceae_gen_Incertae_sedis	-0.3234	0.0776	0.0000	0.0017	Negative
Dorea	Meyerozyma	-0.3331	0.0884	0.0002	0.0054	Negative
Ruminococcus2	Kodamaea	-0.3333	0.0658	0.0000	0.0001	Negative
Flavonifractor	Cladosporium	-0.3402	0.0825	0.0000	0.0020	Negative
Dorea	Malassezia	-0.3415	0.0786	0.0000	0.0009	Negative
Flavonifractor	Aspergillaceae_gen_Incertae_sedis	-0.3426	0.0664	0.0000	0.0001	Negative

Bacterial_Genus	Fungal_Genus	Association_Estimate	Standard_Error	P_value	Q_value	Association_Direction
Lactobacillus	Aspergillaceae_gen_Incertae_sedis	-0.3463	0.1019	0.0007	0.0142	Negative
Lactobacillus	Geotrichum	-0.3496	0.1014	0.0006	0.0125	Negative
Butyricicoccus	Basidiomycota_gen_Incertae_sedis	-0.3496	0.1074	0.0011	0.0199	Negative
Clostridium_XI	Aspergillaceae_gen_Incertae_sedis	-0.3497	0.1039	0.0008	0.0152	Negative
Clostridium_IV	Sordariomycetes_gen_Incertae_sedis	-0.3531	0.1102	0.0014	0.0229	Negative
Clostridium_XVIII	Pichia	-0.3545	0.0900	0.0001	0.0035	Negative
Megasphaera	Sporidiobolales_gen_Incertae_sedis	-0.3596	0.1212	0.0030	0.0388	Negative
Clostridium_XlVb	Kodamaea	-0.3633	0.1154	0.0016	0.0259	Negative
Barnesiella	Talaromyces	-0.3863	0.1083	0.0004	0.0090	Negative
Dorea	Kurtzmaniella	-0.3887	0.1285	0.0025	0.0340	Negative
Butyricicoccus	Exophiala	-0.3895	0.1109	0.0004	0.0106	Negative
Lactobacillus	Debaryomyces	-0.3956	0.1243	0.0015	0.0238	Negative
Anaerostipes	Herpotrichiellaceae_gen_Incertae_sedis	-0.4013	0.1108	0.0003	0.0080	Negative

Bacterial_Genus	Fungal_Genus	Association_Estimate	Standard_Error	P_value	Q_value	Association_Direction
Barnesiella	Malassezia	-0.4238	0.1097	0.0001	0.0043	Negative
Paraprevotella	Cladosporium	-0.4314	0.1276	0.0007	0.0149	Negative
Lactobacillus	Wallemia	-0.4374	0.1299	0.0008	0.0152	Negative
Clostridium_XlVb	Malassezia	-0.4504	0.1258	0.0003	0.0087	Negative
Haemophilus	Candida	-0.4689	0.1613	0.0037	0.0446	Negative
Haemophilus	Lodderomyces	-0.4892	0.1653	0.0031	0.0396	Negative
Barnesiella	Aspergillaceae_gen_Incertae_sedis	-0.4903	0.1278	0.0001	0.0046	Negative
Clostridium_XlVb	Wickerhamomyces	-0.4917	0.1121	0.0000	0.0008	Negative
Haemophilus	Ascomycota_gen_Incertae_sedis	-0.4922	0.1339	0.0002	0.0070	Negative
Dorea	Sordariomycetes_gen_Incertae_sedis	-0.5084	0.1314	0.0001	0.0042	Negative
Clostridium_XlVb	Aspergillaceae_gen_Incertae_sedis	-0.5107	0.1130	0.0000	0.0005	Negative
Collinsella	Eremothecium	-0.5141	0.1527	0.0008	0.0152	Negative
Collinsella	Monascus	-0.5277	0.1635	0.0013	0.0214	Negative
Desulfovibrio	Erythrobasidium	-0.5307	0.1772	0.0027	0.0365	Negative

Bacterial_Genus	Fungal_Genus	Association_Estimate	Standard_Error	P_value	Q_value	Association_Direction
Butyricicoccus	Coprinellus	-0.5318	0.1388	0.0001	0.0046	Negative
Collinsella	Erythrobasidium	-0.5382	0.0999	0.0000	0.0000	Negative
Butyricicoccus	Tausonia	-0.5428	0.1130	0.0000	0.0002	Negative
Clostridium_XVIII	Phaeosphaeria	-0.5433	0.1724	0.0016	0.0256	Negative
Desulfovibrio	Aspergillaceae_gen_Incertae_sedis	-0.5497	0.1160	0.0000	0.0003	Negative
Alloprevotella	Rhizopus	-0.5530	0.1571	0.0004	0.0104	Negative
Alistipes	Colletotrichum	-0.5590	0.1567	0.0004	0.0090	Negative
Butyricicoccus	Sordariomycetes_gen_Incertae_sedis	-0.5634	0.1711	0.0010	0.0180	Negative
Sutterella	Paecilomyces	-0.5673	0.1736	0.0011	0.0193	Negative
Collinsella	Sordariomycetes_gen_Incertae_sedis	-0.5763	0.1380	0.0000	0.0017	Negative
Barnesiella	Cystobasidium	-0.5824	0.1345	0.0000	0.0009	Negative
Anaerostipes	Rhizopus	-0.5829	0.1235	0.0000	0.0003	Negative
Fusobacterium	Sordariomycetes_gen_Incertae_sedis	-0.5843	0.1368	0.0000	0.0012	Negative
Clostridium_IV	Phaeosphaeria	-0.5871	0.2043	0.0041	0.0476	Negative

Bacterial_Genus	Fungal_Genus	Association_Estimate	Standard_Error	P_value	Q_value	Association_Direction
Parasutterella	Fusarium	-0.5893	0.1273	0.0000	0.0004	Negative
Klebsiella	Onygenales_gen_Incertae_sedis	-0.5955	0.1643	0.0003	0.0080	Negative
Haemophilus	Coprinellus	-0.5962	0.1837	0.0012	0.0203	Negative
Flavonifractor	Psathyrellaceae_gen_Incertae_sedis	-0.5976	0.1592	0.0002	0.0055	Negative
Faecalicoccus	Malassezia	-0.6031	0.1806	0.0008	0.0163	Negative
Acetanaerobacterium	Psathyrellaceae_gen_Incertae_sedis	-0.6076	0.2012	0.0025	0.0344	Negative
Collinsella	Coprinopsis	-0.6130	0.1974	0.0019	0.0286	Negative
Megasphaera	Neovaginatispora	-0.6143	0.1581	0.0001	0.0041	Negative
Bilophila	Erythrobasidium	-0.6176	0.1742	0.0004	0.0096	Negative
Alloprevotella	Occultifur	-0.6192	0.1094	0.0000	0.0000	Negative
Dorea	Sebacina	-0.6253	0.1599	0.0001	0.0039	Negative
Clostridium_sensu_stricto	Saccharomycetales_gen_Incertae_sedis	-0.6271	0.1922	0.0011	0.0195	Negative
Clostridium_XVIII	Xerogeomyces	-0.6298	0.2110	0.0028	0.0372	Negative
Dorea	Didymocyrtis	-0.6326	0.1330	0.0000	0.0003	Negative

Bacterial_Genus	Fungal_Genus	Association_Estimate	Standard_Error	P_value	Q_value	Association_Direction
Megamonas	Phallus	-0.6334	0.1255	0.0000	0.0001	Negative
Bilophila	Coprinopsis	-0.6353	0.1745	0.0003	0.0077	Negative
Barnesiella	Paecilomyces	-0.6424	0.1477	0.0000	0.0009	Negative
Romboutsia	Erythrobasidium	-0.6465	0.1908	0.0007	0.0146	Negative
Clostridium_XVIII	Eremothecium	-0.6677	0.1944	0.0006	0.0128	Negative
Paraprevotella	Paraxerochrysium	-0.6678	0.1937	0.0006	0.0125	Negative
Dorea	Dipodascaceae_gen_Incertae_sedis	-0.6739	0.1061	0.0000	0.0000	Negative
Propionibacterium	Dipodascaceae_gen_Incertae_sedis	-0.6829	0.2393	0.0043	0.0496	Negative
Lactobacillus	Pichia	-0.6829	0.1415	0.0000	0.0002	Negative
Butyricicoccus	Coprinopsis	-0.6845	0.2373	0.0039	0.0466	Negative
Romboutsia	Didymocyrtis	-0.6902	0.2081	0.0009	0.0169	Negative
Clostridium_XlVb	Paecilomyces	-0.6907	0.2389	0.0038	0.0463	Negative
Acetanaerobacterium	Sebacina	-0.7047	0.1998	0.0004	0.0102	Negative
Pseudobutyrivibrio	Deconica	-0.7265	0.2499	0.0036	0.0446	Negative

Bacterial_Genus	Fungal_Genus	Association_Estimate	Standard_Error	P_value	Q_value	Association_Direction
Desulfovibrio	Paecilomyces	-0.7292	0.2296	0.0015	0.0241	Negative
Bilophila	Aspergillaceae_gen_Incertae_sedis	-0.7316	0.1829	0.0001	0.0028	Negative
Clostridium_XVIII	Erythrobasidium	-0.7358	0.1739	0.0000	0.0014	Negative
Bilophila	Fusarium	-0.7378	0.1728	0.0000	0.0012	Negative
Barnesiella	Sterigmatomyces	-0.7404	0.1877	0.0001	0.0035	Negative
Parasutterella	Kodamaea	-0.7432	0.1789	0.0000	0.0018	Negative
Acetanaerobacterium	Lodderomyces	-0.7446	0.2128	0.0005	0.0109	Negative
Anaerostipes	Ustilago	-0.7475	0.2149	0.0005	0.0114	Negative
Collinsella	Wickerhamomyces	-0.7506	0.1462	0.0000	0.0001	Negative
Clostridium_XlVb	Saccharomycetales_gen_Incertae_sedis	-0.7547	0.1257	0.0000	0.0000	Negative
Butyricimonas	Cystobasidium	-0.7580	0.2575	0.0032	0.0409	Negative
Butyricicoccus	Nigrospora	-0.7639	0.1961	0.0001	0.0040	Negative
Romboutsia	Nectriaceae_gen_Incertae_sedis	-0.7662	0.1981	0.0001	0.0042	Negative
Barnesiella	Cyberlindnera	-0.7735	0.2580	0.0027	0.0365	Negative

Bacterial_Genus	Fungal_Genus	Association_Estimate	Standard_Error	P_value	Q_value	Association_Direction
Clostridium_XVIII	Kurtzmaniella	-0.7861	0.1860	0.0000	0.0014	Negative
Lactobacillus	Monascus	-0.8060	0.2006	0.0001	0.0027	Negative
Clostridium_XVIII	Dipodascaceae_gen_Incertae_sedis	-0.8160	0.1499	0.0000	0.0000	Negative
Bilophila	Cladosporium	-0.8277	0.2053	0.0001	0.0027	Negative
Desulfovibrio	Coprinopsis	-0.8339	0.1763	0.0000	0.0003	Negative
Barnesiella	Trichosporon	-0.8623	0.2354	0.0002	0.0073	Negative
Clostridium_sensu_stricto	Ustilago	-0.8675	0.2228	0.0001	0.0040	Negative
Bilophila	Exophiala	-0.8826	0.2624	0.0008	0.0152	Negative
Clostridium_XI	Vishniacozyma	-0.8873	0.2522	0.0004	0.0104	Negative
Fusicatenibacter	Millerozyma	-0.8882	0.3090	0.0040	0.0476	Negative
Butyricicoccus	Erythrobasidium	-0.8890	0.2674	0.0009	0.0167	Negative
Haemophilus	Occultifur	-0.9005	0.3097	0.0036	0.0446	Negative
Clostridium_XI	Phaeosphaeria	-0.9034	0.3065	0.0032	0.0407	Negative
Desulfovibrio	Eremothecium	-0.9058	0.2061	0.0000	0.0008	Negative
Butyricicoccus	Nectriaceae_gen_Incertae_s	-0.9091	0.2268	0.0001	0.0028	Negative

Bacterial_Genus	Fungal_Genus	Association_Estimate	Standard_Error	P_value	Q_value	Association_Direction
	edis					
Clostridium_IV	Geotrichum	-0.9318	0.2433	0.0001	0.0046	Negative
Alloprevotella	Sterigmatomyces	-0.9397	0.2127	0.0000	0.0007	Negative
Dorea	Erythrobasidium	-0.9685	0.1733	0.0000	0.0000	Negative
Flavonifractor	Phomatospora	-0.9731	0.3078	0.0016	0.0249	Negative
Desulfovibrio	Clavispora	-0.9748	0.2144	0.0000	0.0004	Negative
Faecalicoccus	Kazachstania	-0.9797	0.3375	0.0037	0.0451	Negative
Butyricicoccus	Phomatospora	-0.9911	0.1997	0.0000	0.0001	Negative
Butyricicoccus	Rhizopus	-1.0099	0.2589	0.0001	0.0040	Negative
Sutterella	Cyberlindnera	-1.0106	0.2968	0.0007	0.0140	Negative
Clostridium_IV	Fusarium	-1.0340	0.2325	0.0000	0.0006	Negative
Odoribacter	Coprinellus	-1.0361	0.3497	0.0031	0.0393	Negative
Coprococcus	Kodamaea	-1.1078	0.3565	0.0019	0.0285	Negative
Faecalicoccus	Clavispora	-1.1149	0.2903	0.0001	0.0046	Negative
Pseudobutyrivibrio	Sebacina	-1.1193	0.3591	0.0018	0.0278	Negative

Bacterial_Genus	Fungal_Genus	Association_Estimate	Standard_Error	P_value	Q_value	Association_Direction
Streptococcus	Ustilago	-1.1339	0.3876	0.0034	0.0427	Negative
Pseudobutyrivibrio	Onygenales_gen_Incertae_sedis	-1.1348	0.3564	0.0015	0.0238	Negative
Romboutsia	Phallus	-1.1610	0.3716	0.0018	0.0273	Negative
Barnesiella	Sporidiobolales_gen_Incertae_sedis	-1.1726	0.3545	0.0009	0.0174	Negative
Haemophilus	Aspergillaceae_gen_Incertae_sedis	-1.1756	0.3115	0.0002	0.0053	Negative
Acidaminococcus	Neosetophoma	-1.1920	0.2967	0.0001	0.0027	Negative
Faecalicoccus	Cyberlindnera	-1.1985	0.3734	0.0013	0.0225	Negative
Sutterella	Didymocyrtis	-1.2227	0.4024	0.0024	0.0329	Negative
Clostridium_sensu_stricto	Kodamaea	-1.2547	0.4059	0.0020	0.0292	Negative
Butyricimonas	Erythrobasidium	-1.2628	0.3645	0.0005	0.0119	Negative
Propionibacterium	Kodamaea	-1.2660	0.4135	0.0022	0.0316	Negative
Clostridium_XVIII	Onygenales_gen_Incertae_sedis	-1.2712	0.2679	0.0000	0.0003	Negative
Odoribacter	Malassezia	-1.2837	0.3666	0.0005	0.0109	Negative
Clostridium_XlVb	Rhizopus	-1.3241	0.3518	0.0002	0.0054	Negative

Bacterial_Genus	Fungal_Genus	Association_Estimate	Standard_Error	P_value	Q_value	Association_Direction
Bilophila	Rhizopus	-1.3522	0.3929	0.0006	0.0127	Negative
Bilophila	Psathyrellaceae_gen_Incertae_sedis	-1.4143	0.3803	0.0002	0.0060	Negative
Lactobacillus	Phallus	-1.4184	0.4611	0.0021	0.0304	Negative
Clostridium_XVIII	Millerozyma	-1.4214	0.4368	0.0011	0.0199	Negative
Bilophila	Xeromyces	-1.4232	0.4226	0.0008	0.0152	Negative
Dorea	Millerozyma	-1.4308	0.4239	0.0007	0.0151	Negative
Romboutsia	Millerozyma	-1.4461	0.4216	0.0006	0.0129	Negative
Akkermansia	Xerogeomyces	-1.4579	0.4181	0.0005	0.0111	Negative
Odoribacter	Saccharomycetales_gen_Incertae_sedis	-1.4851	0.4786	0.0019	0.0286	Negative
Sutterella	Paraxerochrysium	-1.5555	0.4783	0.0011	0.0199	Negative
Bilophila	Kodamaea	-1.6080	0.4882	0.0010	0.0180	Negative
Pseudobutyrivibrio	Nakaseomyces	-1.6247	0.5116	0.0015	0.0241	Negative
Bilophila	Paraxerochrysium	-1.6345	0.5572	0.0034	0.0418	Negative
Odoribacter	Talaromyces	-1.6497	0.4437	0.0002	0.0060	Negative

Bacterial_Genus	Fungal_Genus	Association_Estimate	Standard_Error	P_value	Q_value	Association_Direction
Faecalicoccus	Millerozyma	-1.6830	0.4507	0.0002	0.0058	Negative
Odoribacter	Erythrobasidium	-1.6962	0.4731	0.0003	0.0086	Negative
Dorea	Nakaseomyces	-1.7035	0.5088	0.0008	0.0159	Negative
Clostridium_XVIII	Pleurotus	-1.7449	0.3270	0.0000	0.0000	Negative
Odoribacter	Paecilomyces	-1.7518	0.5869	0.0028	0.0372	Negative
Propionibacterium	Trichosporon	-1.7704	0.6102	0.0037	0.0452	Negative
Clostridium_XI	Psathyrellaceae_gen_Incertae_sedis	-1.7940	0.4817	0.0002	0.0060	Negative
Acetanaerobacterium	Ustilago	-1.7977	0.3734	0.0000	0.0002	Negative
Pseudobutyrivibrio	Pleurotus	-1.8327	0.6380	0.0041	0.0476	Negative
Butyricimonas	Phallus	-1.9060	0.5832	0.0011	0.0193	Negative
Anaerostipes	Clavispora	-1.9065	0.6280	0.0024	0.0329	Negative
Clostridium_XI	Montagnula	-2.1037	0.4557	0.0000	0.0004	Negative
Clostridium_XI	Sterigmatomyces	-2.1169	0.6567	0.0013	0.0216	Negative
Veillonella	Kodamaea	-2.2304	0.5739	0.0001	0.0041	Negative
Clostridium_IV	Millerozyma	-2.2756	0.6747	0.0007	0.0152	Negative

Bacterial_Genus	Fungal_Genus	Association_Estimate	Standard_Error	P_value	Q_value	Association_Direction
Pseudobutyrivibrio	Eremothecium	-2.3339	0.7075	0.0010	0.0178	Negative
Acetanaerobacterium	Auricularia	-2.4363	0.6314	0.0001	0.0043	Negative
Clostridium_XI	Didymosphaeriaceae_gen_Incertae_sedis	-2.5048	0.7820	0.0014	0.0229	Negative
Fusobacterium	Agaricomycetes_gen_Incertae_sedis	-2.6011	0.8569	0.0024	0.0329	Negative
Propionibacterium	Cutaneotrichosporon	-2.6023	0.6925	0.0002	0.0055	Negative
Clostridium_XI	Ustilago	-2.6128	0.8505	0.0021	0.0307	Negative
Clostridium_XI	Naganishia	-2.6458	0.5813	0.0000	0.0004	Negative
Butyricimonas	Nakaseomyces	-2.9739	0.7841	0.0001	0.0051	Negative
Haemophilus	Cladosporium	-3.1026	0.5609	0.0000	0.0000	Negative
Lactobacillus	Wickerhamiella	-3.2421	0.6548	0.0000	0.0001	Negative
Faecalicoccus	Nakaseomyces	-3.2814	0.6112	0.0000	0.0000	Negative
Sutterella	Saccharomycopsis	-3.3907	1.1361	0.0028	0.0372	Negative
Acetanaerobacterium	Clavispora	-3.4615	0.9608	0.0003	0.0084	Negative
Propionibacterium	Millerozyma	-3.6501	1.1607	0.0017	0.0261	Negative

Bacterial_Genus	Fungal_Genus	Association_Estimate	Standard_Error	P_value	Q_value	Association_Direction
Haemophilus	Colletotrichum	-3.8108	0.8710	0.0000	0.0008	Negative
Haemophilus	Rhizopus	-3.9948	1.1422	0.0005	0.0109	Negative
Acidaminococcus	Ganoderma	-4.3883	1.1661	0.0002	0.0054	Negative
Clostridium_XI	Millerozyma	-5.0284	1.4455	0.0005	0.0114	Negative
Butyricimonas	Millerozyma	-5.1385	1.5726	0.0011	0.0193	Negative
Acidaminococcus	Monascus	-5.1994	1.3698	0.0001	0.0051	Negative
Pseudobutyrivibrio	Cladosporium	-5.4979	1.7186	0.0014	0.0230	Negative
Sutterella	Millerozyma	-5.6520	1.6981	0.0009	0.0167	Negative
Fusobacterium	Auricularia	-5.7235	1.4191	0.0001	0.0027	Negative
Sutterella	Phallus	-7.5146	2.2662	0.0009	0.0169	Negative
Pseudobutyrivibrio	Phallus	-7.6468	2.1191	0.0003	0.0083	Negative

Qvalue is the *p*-value corrected for FDR.

Microbiota excluded from DII/LE8-related taxa analysis.

Table S5B. Significant bacterial–fungal associations identified based on DIABLO-selected features.

from	to	weight	*p*_value	abs_weight

Butyricimonas	Fungi_gen_Incertae_sedis	0.225	0.000	0.225
Butyricimonas	Aspergillus	0.190	0.001	0.190
Paraprevotella	Acremonium	0.186	0.002	0.186
Desulfovibrio	Saccharomyces	0.166	0.005	0.166
Akkermansia	Saccharomyces	0.146	0.014	0.146
Acetanaerobacterium	Saccharomycetales_gen_Incertae_sedis	0.143	0.016	0.143
Desulfovibrio	Aspergillus	0.142	0.016	0.142
Lactobacillus	Saccharomyces	0.142	0.017	0.142
Acetanaerobacterium	Aspergillus	0.139	0.019	0.139
Sutterella	Dipodascaceae_gen_Incertae_sedis	0.135	0.022	0.135
Desulfovibrio	Fungi_gen_Incertae_sedis	0.129	0.030	0.129
Prevotella	Fusarium	0.127	0.032	0.127
Paraprevotella	Kazachstania	0.126	0.033	0.126
Acetanaerobacterium	Fungi_gen_Incertae_sedis	0.120	0.042	0.120
Acetanaerobacterium	Debaryomyces	0.119	0.044	0.119
Alloprevotella	Fungi_gen_Incertae_sedis	0.119	0.044	0.119
Barnesiella	Fusarium	0.117	0.048	0.117
Veillonella	Fusarium	-0.119	0.044	0.119
Clostridium_sensu_stricto	Talaromyces	-0.121	0.041	0.121
Butyricimonas	Meyerozyma	-0.121	0.041	0.121
Propionibacterium	Penicillium	-0.123	0.038	0.123
Propionibacterium	Wallemia	-0.124	0.036	0.124
Acetanaerobacterium	Talaromyces	-0.125	0.035	0.125
Butyricimonas	Rhodotorula	-0.126	0.034	0.126
Dialister	Debaryomyces	-0.129	0.029	0.129
Propionibacterium	Rhodotorula	-0.129	0.029	0.129

Propionibacterium	Candida	-0.131	0.026	0.131
Dialister	Aspergillus	-0.134	0.024	0.134
Veillonella	Penicillium	-0.138	0.020	0.138
Parasutterella	Ascomycota_gen_Incertae_sedis	-0.139	0.019	0.139
Lactobacillus	Penicillium	-0.140	0.018	0.140
Klebsiella	Aspergillus	-0.142	0.016	0.142
Klebsiella	Fungi_gen_Incertae_sedis	-0.144	0.015	0.144
Propionibacterium	Aspergillus	-0.145	0.014	0.145
Dialister	Pichia	-0.148	0.012	0.148
Propionibacterium	Ascomycota_gen_Incertae_sedis	-0.152	0.010	0.152
Acidaminococcus	Fungi_gen_Incertae_sedis	-0.164	0.005	0.164
Acidaminococcus	Fusarium	-0.167	0.005	0.167
Lactobacillus	Wallemia	-0.167	0.005	0.167
Lactobacillus	Fungi_gen_Incertae_sedis	-0.168	0.005	0.168
Akkermansia	Kazachstania	-0.170	0.004	0.170
Parasutterella	Exophiala	-0.171	0.004	0.171
Alloprevotella	Pichia	-0.172	0.004	0.172
Propionibacterium	Fungi_gen_Incertae_sedis	-0.179	0.002	0.179
Veillonella	Aspergillus	-0.192	0.001	0.192
Propionibacterium	Dipodascaceae_gen_Incertae_sedis	-0.209	0.000	0.209
Veillonella	Fungi_gen_Incertae_sedis	-0.246	0.000	0.246

Supplementary Table S6. FUNGuild-based fungal ecological guilds across DII and CVH groups

taxon	phylum_genus	trophic_mode	guild	confidence_ranking	HDII	MDII	LDII	HCVH	MCVH	LCVH	*p*_value_DII	FDR_DII	*p*_value_CVH	FDR_CVH	interpretation_category
Animal Parasite	Ascomycota	Pathotroph-Saprotroph-Symbiotroph	Animal Parasite	Probable	10.06	7.23	6.34	7.12	8.28	4.33	0.0086	0.162	0.2143	0.8038	mixed
Animal Pathogen	Ascomycota	Pathotroph-Saprotroph-Symbiotroph	Animal Pathogen	Probable	29.51	22.9	21.14	20.66	24.23	30.47	0.0108	0.162	0.7952	0.9301	mixed
Nematophagous	Basidiomycota	Pathotroph-Saprotroph	Nematophagous	Probable	0	0.23	1.08	2.03	0.31	0	0.026	0.2232	0.0918	0.4592	mixed
Animal Symbiotroph	Ascomycota	Pathotroph-Saprotroph-Symbiotroph	Animal Symbiotroph	Probable	4.04	2.67	2.46	1.93	3.32	1.5	0.0298	0.2232	0.3927	0.8763	mixed
Epiphyte	Ascomycota	Pathotroph-Saprotroph-Symbiotroph	Epiphyte	Probable	14.28	12.01	9.01	8.12	11.03	22.42	0.0438	0.263	0.0483	0.3626	mixed
Endomycorrhizal	Basidiomycota	Pathotroph-Saprotroph-Symbiotroph	Endomycorrhizal	Possible	0	0.02	0.04	0	0.02	0.01	0.0777	0.3886	0.529	0.9301	mixed

taxon	phylum_genus	trophic_mode	guild	confidence_ranking	HDII	MDII	LDII	HCVH	MCVH	LCVH	*p*_value_DII	FDR_DII	*p*_value_CVH	FDR_CVH	interpretation_category
Endosymbiont	Ascomycota	Pathotroph-Saprotroph-Symbiotroph	Endosymbiont	Probable	13.17	10.79	8.38	7.53	10.1	20.4	0.0924	0.3962	0.0063	0.103	mixed
Lichen Parasite	Ascomycota	Pathotroph-Saprotroph-Symbiotroph	Lichen Parasite	Possible	0.66	0.9	3.39	0.44	1.95	0.27	0.1585	0.5943	0.448	0.8763	mixed
Endophyte	Ascomycota	Pathotroph-Saprotroph-Symbiotroph	Endophyte	Probable	29.61	26.2	25.9	27.8	26.55	32.85	0.311	0.8484	0.6606	0.9301	mixed
Lichenized	Ascomycota	Symbiotroph	Lichenized	Highly Probable	0.01	0.01	0	0.01	0	0.03	0.3465	0.8484	0.0818	0.4592	symbiotroph
Ericoid Mycorrhizal	Ascomycota	Pathotroph-Saprotroph-Symbiotroph	Ericoid Mycorrhizal	Probable	0.01	0.03	0	0	0.02	0	0.3538	0.8484	0.4674	0.8763	mixed
Fish Parasite	Ascomycota	Pathotroph-Saprotroph	Fish Parasite	Probable	0	0	0	0.01	0	0	0.3737	0.8484	0.0069	0.103	mixed
Pollen Saprotroph	Chytridiomycota	Pathotroph-Saprotroph	Pollen Saprotroph	Probable	0	0	0.03	0	0.01	0	0.3794	0.8484	0.8991	0.9301	mixed

taxon	phylum_genus	trophic_mode	guild	confidence_ranking	HDII	MDII	LDII	HCVH	MCVH	LCVH	*p*_value_DII	FDR_DII	*p*_value_CVH	FDR_CVH	interpretation_category
Leaf Saprotroph	Basidiomycota	Pathotroph-Saprotroph-Symbiotroph	Leaf Saprotroph	Possible	0.08	0.04	0.18	0.04	0.12	0.03	0.4478	0.8484	0.8678	0.9301	mixed
Insect Pathogen	Ascomycota	Pathotroph-Symbiotroph	Insect Pathogen	Possible	1.53	0.82	0.02	0	0.76	1.77	0.4527	0.8484	0.5644	0.9301	mixed
Algal Parasite	Ascomycota	Pathotroph-Saprotroph	Algal Parasite	Probable	0.19	0.07	0.02	0.03	0.1	0.08	0.459	0.8484	0.8146	0.9301	mixed
Fungal Parasite	Ascomycota	Pathotroph-Saprotroph-Symbiotroph	Fungal Parasite	Highly Probable	5.89	6.54	5.09	6.63	5.83	5.05	0.4807	0.8484	0.4158	0.8763	mixed
Bryophyte Parasite	Basidiomycota	Pathotroph-Saprotroph-Symbiotroph	Bryophyte Parasite	Possible	0.27	0.1	0.2	0.08	0.21	0.11	0.527	0.8579	0.7215	0.9301	mixed
Orchid Mycorrhizal	Basidiomycota	Symbiotroph	Orchid Mycorrhizal	Possible	0.06	0.06	0.06	0.02	0.07	0.04	0.5638	0.8579	0.8866	0.9301	symbiotroph
Plant Pathogen	Ascomycota	Pathotroph-Saprotroph-Symbiotroph	Plant Pathogen	Probable	10.89	12.91	14.21	14.06	12.96	8.72	0.5719	0.8579	0.19	0.8038	mixed
Root Associated Biotroph	Basidiomycota	Symbiotroph	Root Associated Biotroph	Possible	0.06	0.06	0.06	0.02	0.06	0.04	0.6043	0.8633	0.8875	0.9301	symbiotroph

taxon	phylum_genus	trophic_mode	guild	confidence_ranking	HDII	MDII	LDII	HCVH	MCVH	LCVH	*p*_value_DII	FDR_DII	*p*_value_CVH	FDR_CVH	interpretation_category
Ectomycorrhizal	Basidiomycota	Pathotroph-Saprotroph-Symbiotroph	Ectomycorrhizal	Possible	1.1	0.15	0.52	0.14	0.61	0.76	0.6457	0.8805	0.7023	0.9301	mixed
Plant Parasite	Basidiomycota	Pathotroph-Saprotroph-Symbiotroph	Plant Parasite	Possible	0.08	0.05	0.21	0.04	0.13	0.03	0.7259	0.8847	0.792	0.9301	mixed
Litter Saprotroph	Mortierellomycota	Saprotroph-Symbiotroph	Litter Saprotroph	Possible	0.02	0.03	0.02	0.05	0.02	0	0.7563	0.8847	0.277	0.8763	mixed
Soil Saprotroph	Mortierellomycota	Saprotroph-Symbiotroph	Soil Saprotroph	Possible	0.02	0.03	0.03	0.05	0.02	0.08	0.7563	0.8847	0.3404	0.8763	mixed
Clavicipitaceous Endophyte	Ascomycota	Pathotroph-Symbiotroph	Clavicipitaceous Endophyte	Possible	1.58	0.8	0.08	0.03	0.78	1.86	0.7687	0.8847	0.9826	0.9826	mixed
Arbuscular Mycorrhizal	Glomeromycota	Symbiotroph	Arbuscular Mycorrhizal	Highly Probable	0.03	0.01	0	0.01	0.01	0.09	0.7962	0.8847	0.4104	0.8763	symbiotroph
Plant Saprotroph	Ascomycota	Pathotroph-Saprotroph-Symbiotroph	Plant Saprotrophe	Probable	12.51	10.34	14.09	15.45	12.29	9.15	0.8684	0.9305	0.4206	0.8763	mixed

taxon	phylum_genus	trophic_mode	guild	confidence_ranking	HDII	MDII	LDII	HCVH	MCVH	LCVH	*p*_value_DII	FDR_DII	*p*_value_CVH	FDR_CVH	interpretation_category
Wood Saprotroph	Ascomycota	Pathotroph-Saprotroph-Symbiotroph	Wood Saprotroph	Probable	15.85	16.3	19.92	26.58	16.77	13.43	0.9593	0.9603	0.0446	0.3626	mixed
Dung Saprotroph	Ascomycota	Pathotroph-Saprotroph-Symbiotroph	Dung Saprotroph	Highly Probable	3.32	4.37	4.77	5.64	4.14	2.78	0.9603	0.9603	0.6026	0.9301	mixed
